# Water-Network-Triggered Breakdown: Multiscale Theoretical Insights into PET Hydrolysis under Working Conditions

**DOI:** 10.1021/acs.jpcb.6c00486

**Published:** 2026-04-28

**Authors:** Shuangxiu Max Ma, Changlong Zou, Bhavik R. Bakshi, Li-Chiang Lin

**Affiliations:** † William G. Lowrie Department of Chemical and Biomolecular Engineering, 2647 The Ohio State University, Columbus, Ohio 43210, United States; ‡ School for Engineering of Matter, Transport and Energy, 7864 Arizona State University, Tempe, Arizona 85281, United States; § School of Sustainability, Arizona State University, Tempe, Arizona 85281, United States; ∥ School of Complex Adaptive Systems, Arizona State University, Tempe, Arizona 85281, United States; ⊥ Department of Chemical Engineering, 33561 National Taiwan University, Taipei 10617, Taiwan

## Abstract

Polyethylene terephthalate (PET) undergoes depolymerization in the presence of active water, a principle that is widely utilized in innovative chemical-recycling reactors. However, the intertwined effects of water sorption, nanoscale reconfiguration, and the energetics of ester bonds remain predominantly unquantified. Here, a multiscale workflow is developed that links sorption thermodynamics to reaction kinetics by combining molecular simulations with density functional theory (DFT). Simulations quantify PET water uptake, swelling, and water mobility under reactor-relevant conditions and reveal a clear hydration threshold in the polymer phase: when hydration remains below this level, water access is limited and chain scission events are rare; once hydration exceeds it, interconnected water clusters form and hydrolysis accelerates sharply. Above the threshold, end-initiated “peeling” becomes dominant, rapidly producing MHET/BHET and ultimately terephthalic acid (TPA) and ethylene glycol (EG) as reactions proceed within these active water domains. DFT further explains this rate jump: extended hydrogen-bond networks in clustered water enable proton-relay assistance, which stabilizes the tetrahedral intermediate and lowers the hydrolysis barrier compared with attack by an isolated water molecule. Incorporating these barrier reductions together with simulation-derived, loading-dependent water mobility into a kinetic model reproduces both the acceleration at high water availability and the slowdown as water is depleted, clarifying when uptake, transport, or intrinsic chemistry is rate-determining. Overall, the results provide a quantitatively predictive, theory-based description of PET hydrolysis under realistic reactor conditions and translate directly into design principles for tunable, high-efficiency polyester depolymerization.

## Graphical abstract

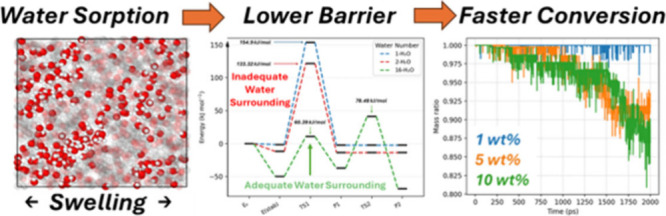

## 1 Introduction

Polyethylene terephthalate (PET) underpins modern packaging and textiles, with approximately 70 million tons produced annually, and global demand continues to rise.^1^ Yet fewer than 20% of discarded PET items are ever recycled; most accumulate in landfills or the environment, contributing to the 6,300 Mt global plastic waste backlog.^2^ Mechanical reprocessing cannot close this loop because each melt step degrades molecular weight, leading to the limitation of the reproduce-reuse cycle.^3^ Chemical recycling offers a route back to virgin-grade feedstocks. Among the various depolymerization pathways, including glycolysis, methanolysis, pyrolysis, enzyme-catalyzed reactions, and hydrolysis, aqueous hydrolysis stands out for three reasons. First, it directly regenerates the two monomers used in today’s TPA/EG melt processes, enabling closed-loop reuse without the need for complex transesterification.^4^ Second, the only stoichiometric reagent is water; acids, bases, or heterogeneous catalysts merely accelerate a reaction that is thermodynamically favored by the presence of excess H_2_O, which is tunable and environmentally friendly.^5^ Third, hydrolysis tolerates mixed or colored PET streams that plague mechanical and glycolytic routes with industrial demonstrations now achieving ≥80% depolymerization within 10 h.^6^

However, the hydrolysis of PET in a working environment presents unresolved fundamental questions at the interface of polymer physics and chemistry. Specifically, despite recent advances in PET depolymerization, our molecular-level understanding of water uptake, nanoscale structuring, and ester bond reactivity remains limited.^7^ Water sorption in PET is intrinsically low and highly sensitive to morphology with crystallinity as low as ∼25% dramatically impedes water ingress, restricting hydrolysis to amorphous domains.^8,9^ FTIR studies reveal that penetrant water in PET does not simply form a bulk-like network; instead, water’s hydrogen-bond structure is heavily perturbed by specific polymer interactions and constrained free volume.^10^ Such nanoscale structuring couples directly to reactivity: hydrolysis initiates preferentially in glassy or amorphous regions where water can access ester linkages.^11^ Experiments indeed suggest a strongly coupled reaction–diffusion mechanism, where transport of water and chemical bond cleavage feed back into each other. For example, if hydrolysis outpaces water diffusion, degradation becomes confined to surface layers; conversely, sufficiently rapid water uptake yields more homogeneous bulk erosion.^8^ However, current knowledge of sorption kinetics, diffusion pathways, and bond scission rates in PET is either unquantified or fragmented with numerous empirical kinetic models proposed in isolation. A unified picture that links polymer microstructure, water distribution, and ester bond reactivity under hydrothermal conditions is still lacking.

Direct in situ observation of water uptake, clustering, and bond cleavage in PET under hydrothermal conditions is extremely challenging. In situ characterization techniques require a more stable chemical environment and fail to capture the resolution necessary to observe the atomistic-level behavior of low amounts of water within the polymer structures.^12^ Consequently, molecular simulations have become indispensable for probing these nanoscale processes (including adsorption, transport and reaction). Grand-canonical Monte Carlo (GCMC) and related test-particle insertion techniques (e.g., the Widom insertion method) are widely used to model water sorption in polymer structures.^13–18^ GCMC simulations sample the addition/removal of water molecules at a fixed chemical potential, yielding equilibrium uptakes and insights into where water molecules preferentially reside in the polymer. Such Monte Carlo methods can capture the thermodynamics of sorption including the propensity for water to occupy free volume sites or form clusters. However, GCMC is implemented at fixed volume, so polymer swelling and the associated changes in free volume are not captured. To complement GCMC, classical molecular dynamics (MD) is applied to the same polymer–water compositions in the isothermal–isobaric ensemble (i.e., NPT ensemble), allowing the simulation cell to expand and the polymer matrix to relax, thereby quantifying swelling, water clustering, and mobility under the corresponding thermodynamic state. In this workflow, Widom insertion provides the equilibrium water uptake at a specified temperature and water activity, while MD translates that uptake into transport-relevant properties—most importantly, the loading-dependent diffusivity and the emergence (or absence) of connected water domains.^19^ Turning from transport to chemistry, hydrolysis requires ester-bond scission. Because classical MD enforces a fixed molecular topology (no bond making/breaking), it cannot capture the reaction; reactive simulations and electronic structure calculations are therefore used to resolve the mechanism and barriers. To this end, reactive MD using a reactive force field (ReaxFF) circumvents that limitation by allowing chemical bonds in the polymer system to break and form during the simulation.^20,21^ ReaxFF-based MD has been applied to PET systems to elucidate degradation pathways, successfully capturing spontaneous ester bond cleavage and subsequent reactions.^22^ The reaction mechanisms and activation energies predicted by ReaxFF molecular dynamics (MD) show good agreement with experimentally measured kinetics, increasing confidence that the model captures the underlying chemistry with sufficient realism. Integrating water adsorption/uptake predictions with ReaxFF MD enables a quantitative assessment of how local water content reshapes the reactive region, moving beyond the oversimplified water–polymer interface models used in prior studies.^23^ To further refine the mechanistic picture and associated energetics, representative elementary pathways can be extracted from the ReaxFF-derived reaction landscape and evaluated using density functional theory (DFT). DFT calculations on well-defined model systems with oligomeric ester fragments coordinated with explicit water molecules can isolate intrinsic hydrolysis steps, resolve key transition states, and provide high-fidelity activation energies. DFT can provide precise activation barriers and identify transition states for ester bond cleavage, serving as a benchmark for force-field simulations.^15^ However, these calculations remain restricted to nanoscopic models and short time scales, and thus cannot by themselves describe water uptake, long-time diffusion, or cooperative dynamics in bulk PET; such macroscopic behavior should be captured by the force field simulations and a continuum kinetic model.^24^ Building on those needs, prior computational work has often parametrized uptake, transport, and chemistry independently, for example, treating sorption without polymer relaxation, assigning diffusion mainly from temperature while neglecting hydration effects, or modeling reactivity without the structural changes induced by swelling—so the feedback among water uptake, clustering, relaxation, and bond cleavage under operating conditions remain unclear. In this study, Widom insertion, ReaxFF MD, and DFT are integrated into a single workflow and embedded in a continuum model to resolve these coupled processes.

Accordingly, a multiscale framework is constructed for PET hydrolysis under reactor-relevant hydrothermal conditions. Reactor temperature–pressure conditions are first mapped to the water chemical potential (μ) (and activity), and Widom insertion is used to determine the corresponding equilibrium water uptake and swelling state of amorphous PET. The resulting μ-consistent swollen configurations then serve as shared initial states for (i) quantifying water structures and deriving a water-loading-dependent diffusivity model and (ii) identifying ester-bond hydrolysis pathways and product evolution in the swollen layer. In addition, key elementary steps extracted from the ReaxFF reaction network are refined by DFT to obtain high-fidelity, pathway-specific activation barriers and to resolve proton-transfer motifs within the hydrogen-bond network. Finally, DFT-corrected barriers and loading-dependent diffusivity are embedded in a microkinetic/continuum model to predict bulk PET degradation kinetics under reactor conditions, thereby linking atomistic mechanisms to macroscopic rate behavior with reduced reliance on empirical, decoupled correlations.

## 2 Computational Methods

Figure 1 schematically depicts the multiscale workflow that connects reactor-scale thermodynamic conditions with atomistic PET hydrolysis chemistry. Reactor temperatures and pressures are first converted to a water chemical-potential map using CoolProp with the Peng–Robinson equation of state. This chemical potential fixes the state and density of a dry PET model, which is then equilibrated with ReaxFF MD. Different water loadings are subsequently introduced to generate preswollen PET–water configurations. These swollen PET frameworks are relaxed and propagated in additional ReaxFF simulations, from which we characterize water clustering and diffusion. Water uptake is determined by chemical-potential matching. For a series of ReaxFF-relaxed PET frameworks spanning different preloaded water contents, Widom insertion (RASPA) is used to compute the water chemical potential inside the polymer for each hydration level. The equilibrium uptake (“water uptake limit”) at a given reactor condition is then identified as the hydration level whose polymer-phase chemical potential matches the reservoir chemical potential obtained from the *T*–*p* map. The equilibrium water-uptake limit is identified at the intersection between the environmental water chemical potential (computed with CoolProp) and the simulated chemical-potential–uptake curve. PET–water systems at selected loadings are then used for high-temperature ReaxFF MD that explicitly follows ester-bond cleavage, and the key elementary steps identified in these trajectories are refined with DFT calculations. Finally, the simulated water uptake, DFT-corrected activation barriers, and loading-dependent diffusivities are integrated into a kinetic reaction–diffusion model that predicts PET depolymerization under reactor conditions. The following sections are organized to follow this workflow.

**Figure 1:**
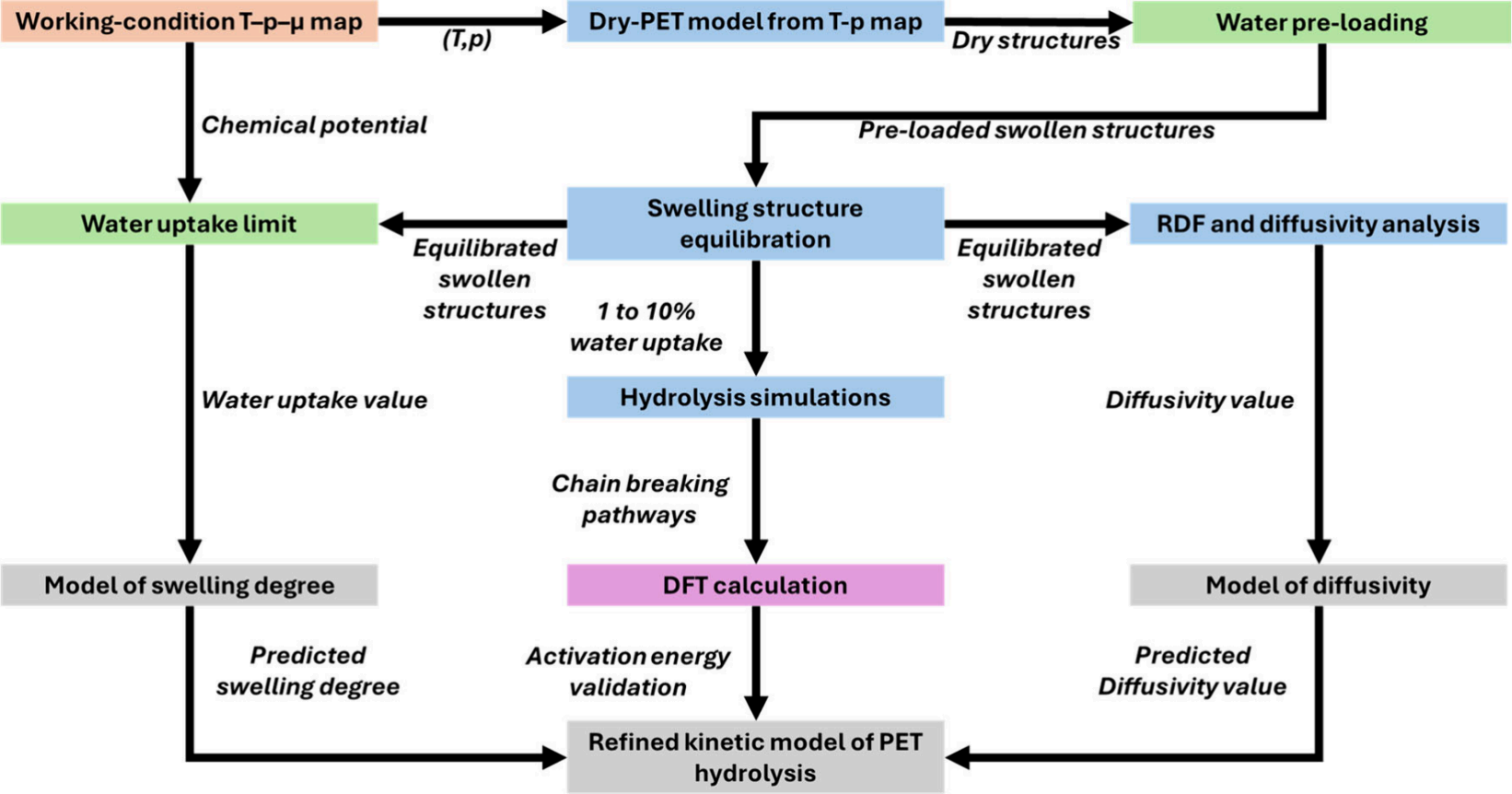
Multiscale workflow for quantitatively predicting PET hydrolysis under reactor conditions. Simulations performed with CoolProp (chemical potential calculation), LAMMPS (ReaxFF MD), RASPA2 (water loading), CP2K (DFT calculation), and numerical modeling are marked with orange, blue, green, purple, and gray frames.

### 2.1 Working-Condition T-p-μ Map

A two-component, isochoric reactor model is constructed using CoolProp^25^ (version 6.4): starting at 300 K, with half of the volume filled with saturated liquid water and the remainder containing dry air at 1 bar. As temperature increases, liquid density and vapor pressure are updated self-consistently, and the noncondensable gas expands in the shrinking headspace. The model, therefore, predicts higher total pressures at slightly elevated T than a single-component Antoine treatment^26^ (see Figure S1), reflecting more realistic constraints when noncondensable gases remain in a fixed volume. Hence, the results of CoolProp are adopted as the working conditions. At each (*T*, *p*) point, the water chemical potential μ is evaluated with the Peng–Robinson equation of state.^27^ The root with the lower μ defines the stable phase. All equations and critical properties, as well as acentric factors, match those used previously for humid-polymer simulations.^13^

### 2.2 Reactive Force Field Details

All molecular dynamics (MD) simulations (Sections 2.3, 2.4, and 2.6) are performed with the open-source LAMMPS package^28^ with the ReaxFF potential,^20^ which was developed and parametrized based on quantum mechanical (QM) data, enabling it to accurately describe bond association and dissociation in reactive systems while maintaining computational efficiency. Such force fields are based on bond orders determined by the distance between pairs of atoms. Various energy contributions can then be accordingly computed, including bond energy (*E*_bond_), overcoordination energy (*E*_over_), under-coordination energy (*E*_under_), valence angle energy (*E*_val_), penalty energy (*E*_pen_), torsion energy (*E*_tors_), conjugation energy (*E*_conj_), and nonbonded van der Waals (*E*_vdWaals_) and Coulomb (*E*_Coulomb_) interactions. In this work, the CHON-2017_Weak force field developed by van Duin et al.^29^ for hydrocarbon/water systems was used to describe PET hydrolysis. This force field has also been successfully utilized in previous MD studies, which accurately describe the interaction of hydrocarbon/water molecules in the condensed phase as benchmarked previously.^30^

### 2.3 Dry-PET Model

A long-chain PET polymer containing 100 monomers (molecular weight = 19200 g/mol) is constructed using the Moltemplate package^31^ and placed in a periodic simulation box to emulate the extended nature of polymer chains. This chain length corresponds to the typical molecular weight range for PET.^32^ The model is first equilibrated for 1 ns in the isothermal–isobaric ensemble (i.e., NPT ensemble at 1 atm and 300 K). The converged bulk density of 1.38 g cm^–3^ falls within the experimental window^33^ of 1.35–1.40 g cm^–3^. Equilibrated dry frameworks are then conditioned (1 ns via NPT simulation) at temperature–pressure points derived from CoolProp (300–600 K, 1–122.8 atm) to obtain density-matched dry structures that serve as the host for the uptake studies below.

### 2.4 Swollen PET–Water Model Construction

The dry PET configurations generated in the previous section (300–600 K; 1–122.8 atm) are used to construct swollen PET–water states at prescribed polymer-phase water contents (0–20 wt %) by inserting a fixed number of water molecules into each framework. These water-loaded configurations are then used as starting points for molecular dynamics equilibration to obtain swollen polymer structures with the appropriate density at each *T*–*p* condition. Specifically, the systems are equilibrated in the NPT ensemble for 1 ns and then in the NVT ensemble for 0.5 ns using the ReaxFF potential described in Section 2.2 (with pressures taken from Section 2.1). From the 0.5 ns NVT trajectories (with water present), water–water cluster statistics and mean-square displacements (MSD) are computed, and diffusion coefficients are obtained from the Einstein relation. To prepare inputs for the chemical potential calculations in the next section, 10 uncorrelated snapshots are extracted from the NVT trajectories; for each snapshot, water molecules are removed and the remaining swollen polymer coordinates are frozen as rigid “swollen-dry” frameworks.

### 2.5 Determining Water Uptake

The chemical-potential-based uptake prediction is then carried out on the swollen-dry frameworks generated in Section 2.4. For each temperature *T* and prescribed loading *w*, the polymer-phase chemical potential of water (μ_ads_) is evaluated by Widom test-particle insertion using the RASPA package.^34^ In each Widom calculation, the swollen PET framework is held rigid and an explicit population of *N* water molecules (corresponding to the target loading *w*) is present in the simulation cell; Widom insertion is performed as a “ghost” probe on top of this fixed-N background. This approach is appropriate here because (i) water loadings remain ≤20 wt %, such that insertion probabilities and chemical potential estimates remain reliable,^13^ and (ii) the PET is markedly more rigid than the flexible framework materials where Widom typically fails.^35,36^ For these calculations, the OPLS-AA^37^ force field is utilized for the PET structures (host), and the TIP4P model^38^ for water molecules (additive). Partial charges for PET are assigned according to the OPLS-AA atom types used in the generated topology (with charge neutrality enforced for the polymer, including end groups), while water employs the standard fixed TIP4P charge distribution (H and M-site charges as defined by TIP4P). The Lennard-Jones (L-J) potentials are truncated and shifted to zero at 12 Å, while the long-range interactions are computed using the Ewald summation method.^39^ It is worth mentioning that a prior investigation conducted by Takuma et al.^40^ demonstrated that the force field used in this study could reasonably describe the water–polymer interactions. Sampling consisted of configurational decorrelation moves of the existing *N* water molecules—translation, rotation, and reinsertion—together with Widom “ghost” insertions; no insertion/deletion (swap) moves of real water molecules are performed. The move ratio is set to 1:1:2:4 (translation/rotation/reinsertion/Widom insertion). Each state is sampled for 5000 equilibration cycles followed by 50000 production cycles, providing converged averages while enabling efficient screening across many hydration levels. The resulting μ_ads_ values are compared with the environmental water chemical potential μ_env_ obtained in Section 2.1 to determine the uptake limit at each temperature.

### 2.6 Hydrolysis Simulations

The systems of 1, 5, and 10 wt % water uptake at 450 K are chosen for simulations of PET degradation with water under the NVT ensemble using the Nosé–Hoover thermostat. For simulating hydrolysis, the systems are first ramped to the desired temperature at a heating rate of 100 K/ps, then held at 1200 K for 1 ns. A time step of 0.1 fs and a temperature damping constant of 100 fs are used in all ReaxFF MD simulations. For each system, five independent simulations are conducted with distinct velocity seeds, and all kinetic quantities and yields reported herein are averages over these simulations. In addition, to obtain a complete reaction landscape, the system with 10 wt % is chosen for an extended 10 ns NVT simulation to reach reaction equilibrium.

### 2.7 DFT Simulations

Density functional theory (DFT) calculations are performed using the Quickstep module in the CP2K package,^41^ employing the PBE exchange-correlation functional^42^ together with Grimme’s D3­(BJ) dispersion correction^43^ for an accurate description of noncovalent interactions. Goedecker–Teter–Hutter (GTH) pseudopotentials^44^ are used in combination with triple-ζ Gaussian basis sets (TZVP-MOLOPT),^45^ and the plane-wave cutoff is set to 400 Ry with a relative cutoff of 55 Ry. Brillouin zone integration is performed with a reciprocal space mesh consisting of only the γ point. Three repeated PET units are linked head-to-tail and placed in the periodic box, and a vacuum of 15 Å is added along the z and y directions to avoid interactions with the adjacent images that are first created. Various numbers of water molecules are introduced into the simulation box to represent diverse water environments, ranging from a single isolated water molecule to a configuration with 16 water molecules. Before DFT geometry optimizations, all structures are first relaxed using ReaxFF under an NVT ensemble for 1 ns, with 5 snapshots from the last 50 ps selected for subsequent DFT calculations. The lowest-energy structure is chosen for the subsequent transition state search. Geometry optimizations are carried out in periodic boundary conditions (PBC) along all three perpendicular directions, using a BFGS algorithm with convergence criteria of 3 × 10^–3^ Å for the maximum displacement and 1 × 10^–3^ Ry/Å for the maximum force. Energy is evaluated at the Γ-point with an SCF convergence criterion of 1× 10^–6^ Ha. The SCF procedure employs a mixing scheme with an alpha value of 0.4, and the multigrid approach is set to five levels to balance accuracy and efficiency. We use the climbing-image nudged-elastic band method (CI-NEB)^46^ to identify transition states. The vibrational analysis is used to further verify transition states; only one imaginary frequency mode indicates that the transition states are the true saddle points.

### 2.8 Kinetic Model

We model neutral hydrolysis of PET in water with a reaction–diffusion framework in which the intrinsic kinetics depend on local hydration and temperature, and internal mass transfer is captured through a Thiele-modulus effectiveness factor. The model is evaluated on a (*T*, *w̅*) grid (temperature *T* in K, mean water loading *w̅*), generating maps of the effective activation barrier *E*_eff_ (*T*, *w̅*) (in Figure S2), the apparent first-order rate constant *k*_app_ (*T*, *w̅*), and the 1 h conversion α (*T*, *w̅*; *t* = 3600 s). We also report the maximum attainable 1 h conversion along an uptake-limit curve with *w*_max_(*T*) within the swollen layer. All energies are in kJ mol^–1^, *R* = 0.008314 kJ mol^–1^ K^–1^, diffusivities in m^2^ s^–1^, and times in seconds. Detailed equations and steps are provided in the Supporting Information, in the “Details for kinetic model” section.

## 3 Results and Discussion

The analysis begins by calculating environmental chemical potential and generating amorphous PET configurations with different water loadings under laboratory-scale water temperatures and the corresponding pressure. The absorbed water structure at the nanoscale and the influence of water uptake on self-diffusion are then examined. ReaxFF trajectories are used to map the hydrolysis network under realistic PET–water configurations and to identify the dominant pathways. DFT calculations refine the key transition states and barriers. Finally, a swelling-aware kinetic model integrates the loading-dependent transport and energetics, reproduces time-resolved conversion data, and enables direct comparison with experimental trends. The subsequent sections of this study adhere to a causal sequence: working conditions, the structure of PET–water, the limitations of water uptake, the chemical reaction, and the kinetics involved.

### 3.1 Working Conditions and Related PET–Water Density

The systematic investigation of PET hydrolysis commences with the delineation of temperature, pressure, and chemical potential pertinent to the operational conditions, followed by the computation of the density of the swollen PET. Figure 2 establishes the thermodynamic envelope explored in this study and quantifies the attendant volumetric response of amorphous PET. Figure 2a shows the seven simulated state points along the saturated-water vapor trajectory and their corresponding environmental chemical potentials, which serve as the thermodynamic reference for the external environment. Over the temperature range of 300–600 K, this reference chemical potential changes by approximately 15 kJ mol^–1^, indicating that the thermodynamic state of the surrounding water varies significantly with operating conditions (*T*, *p*). It is important to distinguish this environmental reference from the insertion chemical potential obtained from Widom insertion calculations, which define the water chemical potential in the PET matrix based on uptake. The final equilibrium water uptake at a specific temperature is determined by locating the uptake level at which the PET-phase chemical potential equals that of the external environment. More details can be found in a later section.

**Figure 2:**
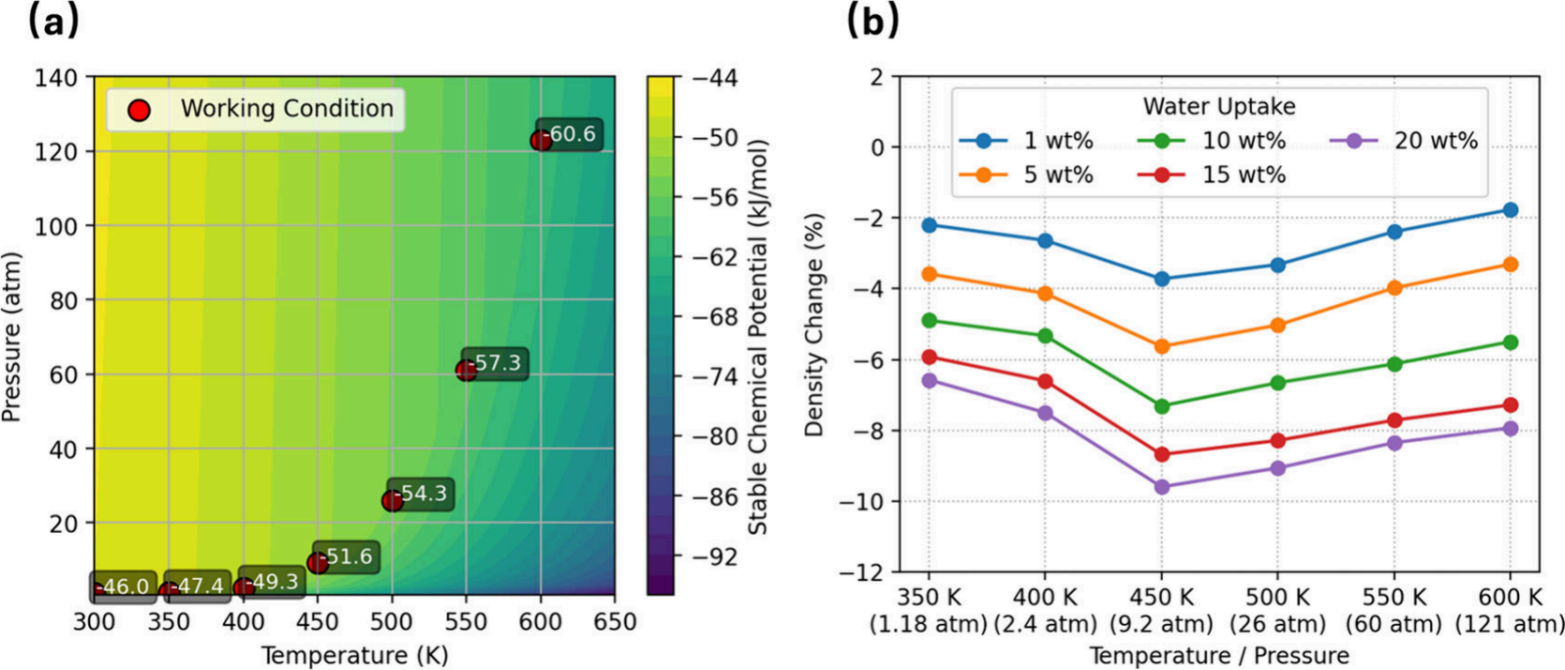
(a) Stable water chemical potential μ­(*T*,*p*) with seven simulation state points highlighted. (b) Results of PET density change from dry polymer versus water uptake.

Figure 2b shows that increasing temperature or water loading in a sealed reactor decreases the PET density relative to the dry polymer. At each *T*–*p* state point, the density drop is nearly linear in water loading between 1 and 20 wt %, indicating that each additional wt % of sorbed water generates a comparable increment of free volume. Above ≈450 K, however, thermal expansion and plasticization are largely offset by the strong compressive stresses in the sealed reactor, so further heating results in only a modest additional density loss. Comparison with dry PET (Figure S3a) confirms that pressure suppresses volume expansion, and the ambient-pressure glass transition near 350 K is no longer a meaningful reference once the sample is confined. The density changes under water uptake, therefore, indicate that, in nonisobaric operation, the system follows a path on the full *T*–*p* surface rather than along a simple isobar. This behavior highlights the need to treat the reactor as a closed, pressure-building system when coupling swelling, diffusion, and hydrolysis kinetics. Relative to ambient conditions (Figure S3b), the overall trend is still a net softening with temperature: at 300 K, 1 wt % water reduces the glassy density by only ∼0.01 g cm^–3^, whereas at 600 K and 122.8 atm the same uptake lowers the density by ∼0.17 g cm^–3^. This larger density loss is consistent with greatly enhanced chain mobility and the creation of free volume at high temperature. The progressive softening with temperature foreshadows the accelerated diffusivity and hydrolysis rates examined in later sections and underscores the need to incorporate water thermodynamics and pressure-dependent swelling into predictive models of water-assisted degradation. It should be noted that these results are reported for amorphous PET. In semicrystalline PET, water uptake is anticipated to occur primarily in amorphous regions, while crystalline domains remain largely inaccessible to water molecules; accordingly, the overall density response may depend on the degree of crystallinity.

### 3.2 RDF and Water Structure

Using the structures constructed under working conditions, the water structure is also analyzed. Radial distribution functions (RDFs) and the distribution of water clusters at 450 K are computed. Figure 3a shows representative snapshots of the simulated system and illustrates how nanoconfined water reorganizes as the uptake in amorphous PET increases from 1 to 20 wt % at 450 K, providing a microscopic basis for the loading-dependent diffusivities discussed later. For each system, a 1 ns NPT equilibration is first performed to ensure that the density reaches equilibrium before the subsequent NVT production run used for RDF analysis. All simulations discussed in Section 3.2 are carried out using ReaxFF.

**Figure 3:**
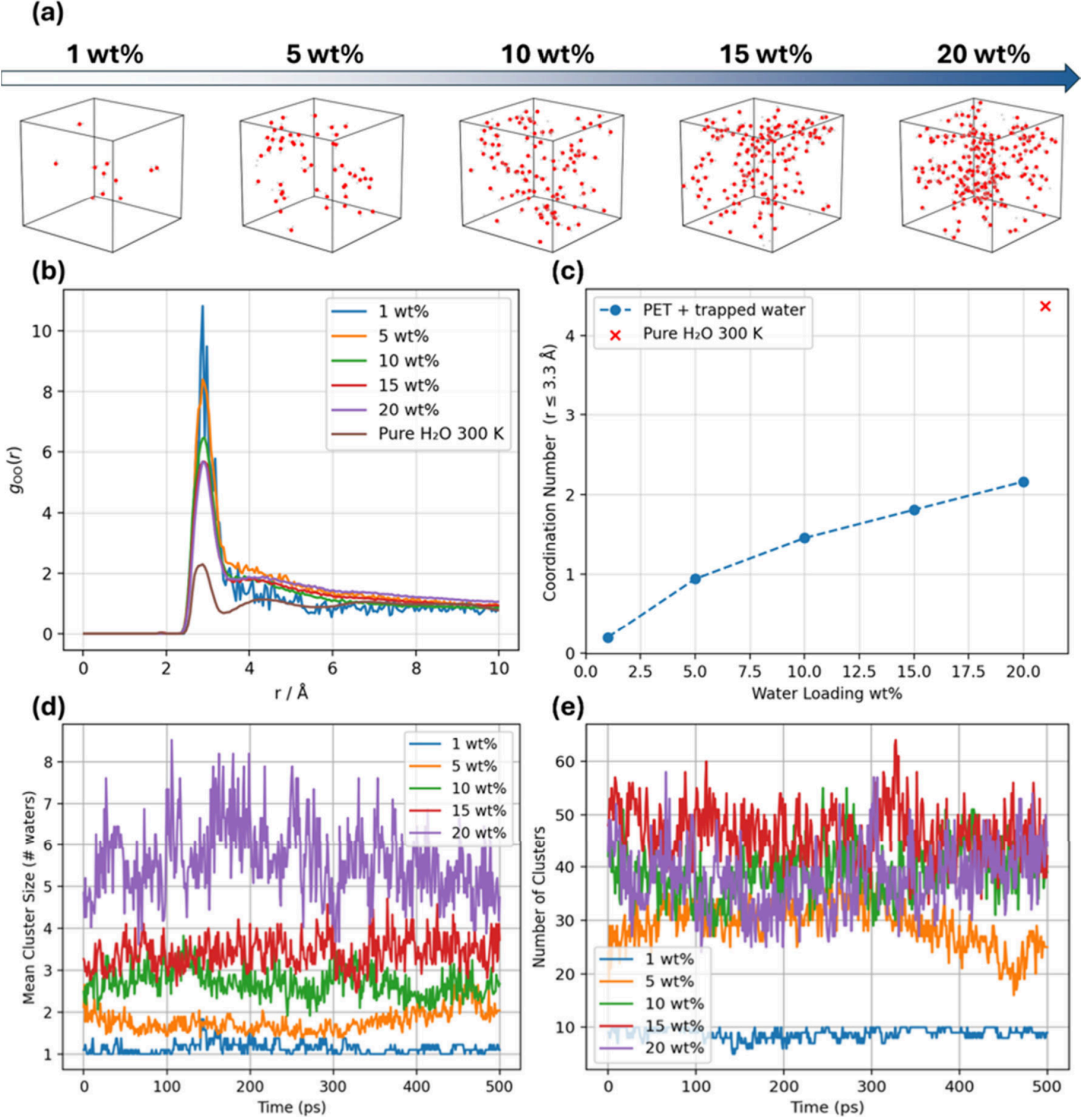
Water structuring inside amorphous PET at 450 K as a function of absorbed water content. (a) Snapshots of water molecules represented by red dots at the end of the simulation. (b) O–O RDF (radial distribution functions), *g*_oo_(*r*), for 1–20 wt % loadings compared with bulk liquid water at 300 K and 1 atm. (c) Average first-shell coordination number using a cutoff radius *r* ≤ 3.3 Å, extracted from the curves in panel (b); the red × marks the bulk-water reference. (d) Time evolution of the mean water-cluster size over 500 ps MD trajectories for different loadings (1–20 wt %). (e) Corresponding number of discrete clusters versus time.

In Figure 3b, the RDF of pure water is first calculated and shows good agreement with the previous study that applied the same force field parameters,^30^ thereby confirming the robustness of the following results for the PET–water system. It is noted that *g*_oo_(*r*) retains the bulk-water first-shell position at ≈2.8 Å, but their amplitudes evolve differently: at the lowest loading, the first peak exceeds a height of 10, while the second and third shells are almost absent. This indicates that the few water molecules present tend to condense into small, tightly bound clusters at the bulk-like O–O contact distance, rather than forming an extended hydrogen-bond network. As the uptake increases, the first-peak amplitude subsides, and a weak second-shell shoulder emerges, indicating the onset of more extended hydrogen-bond topologies, which are consistent with the previously reported water clustering behavior from Okuwaki et al.^47^ These qualitative changes are distilled into the coordination number in Figure 3c: the average first-shell coordination climbs steadily from <0.5 at 1 wt % to ∼2 at 20 wt %, still just half the bulk-water reference (red ×), underscoring that even at the highest loading, water in PET fails to recover the long-range connectivity of the hydrogen-bond network. Meanwhile, as expected, the rising temperature does not distort the instantaneous H-bond distance but steadily erodes network connectivity, replacing larger, more ordered clusters with sparser, transient aggregates (Figure S4).

The cluster statistics in Figure 3d,e describe the same phenomenon from a complementary angle. Water clusters are identified using an oxygen–oxygen distance criterion from Okuwaki et al.^47^ Two water molecules are considered connected if the O–O distance is ≤ 3.5 Å, and a cluster is defined as a group of water molecules linked through this connectivity. The mean cluster size rises almost linearly with loading, reaching 6 to 8 molecules at 20 wt %, while the number of simultaneous clusters increases from just a handful at 1 wt % to ∼40 at intermediate loadings before leveling off as coalescence overtakes nucleation. Collectively, these data reveal a progressive transition, as the loading increases, from sparsely dispersed monomers/dimers to nanometer-sized water clusters that remain topologically isolated; the growth of these clusters enlarges the local free volume and supplies additional hydrogen-bond donors/acceptors, mechanistically explaining the accelerated transport and the greater availability of reactive water that drives the hydrolysis kinetics discussed in the following sections.

### 3.3 Diffusivity

Having established from the RDF analysis that added water reorganizes into increasingly connected clusters, we next quantify how this evolving microstructure translates into molecular mobility. Figure 4a reports mean-square-displacement (MSD) curves at 450 K for five water loadings (1–20 wt %). MSD is calculated with a time-lag/ensemble approach: for every lag time Δ*t*, the trajectory furnishes all displacement vectors *r*(*t* + Δ*t*) – *r*(*t*); their squared magnitudes are ensemble-averaged to obtain ⟨|*r*|^2^⟩(Δ*t*). The log–log scale is adopted in Figure 4a to better resolve differences in MSD behavior across water loadings. At the lowest loading (∼1 wt %), the MSD remains almost an order of magnitude smaller, consistent with individual molecules being pinned by hydrogen bonds or trapped in nanocavities. Since the diffusive window is limited and varies with loading and temperature, diffusivities are obtained from condition-specific fitting windows instead of a single overall interval.

**Figure 4:**
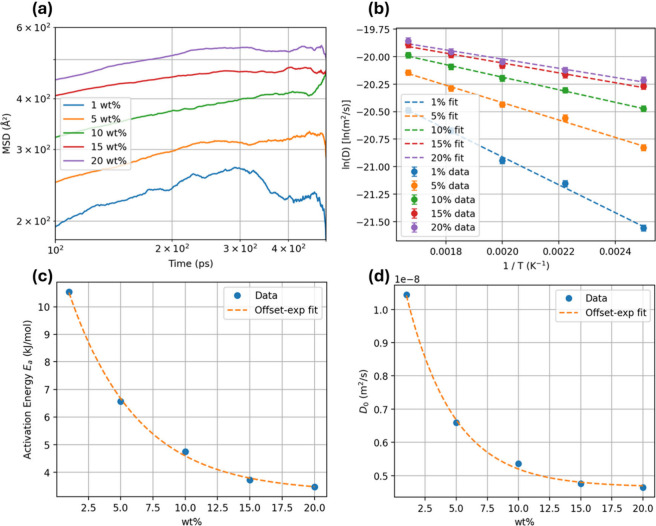
(a) MSD at 450 K for 1–20 wt % water in log–log scale starts from 100 ps. (b) Arrhenius plots of diffusivity; dashed lines represent the corresponding linear fits. (c) Activation energy *E*_a_ extracted from the fits falls sharply with loading. (d) Pre-exponential factor *D*_0_ decreases correspondingly. Orange curves show a common offset-exponential fit used in the kinetic model.

Temperature-resolved simulations (300–600 K; Figure 4b) show classical Arrhenius behavior at each loading, captured by

1 $$D\left(\mathrm{w̅},T\right)=D_{0}\left(\mathrm{w̅}\right)\exp\left(-\frac{E_{D}\left(\mathrm{w̅}\right)}{RT}\right)$$

For each water uptake w̅ , the parameters $E_{D}\left(\mathrm{w̅}\right)$ and $D_{0}\left(\mathrm{w̅}\right)$ are obtained by a least-squares fit of ln *D* versus 1/*T* using all simulated temperatures at that loading; only points for which the MSD exhibited a clear linear diffusive regime are included in the fit. The resulting $E_{D}\left(\mathrm{w̅}\right)$ and $D_{0}\left(\mathrm{w̅}\right)$ values both decrease with water content and are well described by offset–exponential correlations:

2 $$E_{D}\left(\mathrm{w̅}\right)=E_{\infty }+\left(E_{0}-E_{\infty }\right)\exp\left(-k_{E_{a}}\mathrm{w̅}\right)$$

3 $$D_{0}\left(\mathrm{w̅}\right)=D_{0,\infty }+\left(D_{0,0}-D_{0,\infty }\right)exp\left(-k_{D0}\mathrm{w̅}\right)$$

where the constants *E*_∞_, *E*_0_, *k*_*E*_a__, *D*_0,∞_, *D*_0,0_, and *k*_*D*_0__ are obtained by nonlinear least-squares regression over all loadings (fitted values can be found in the Supporting Information). The functional dependence mirrors doping-dependent activation-energy models in semiconductor diffusion, with water acting as a “dopant” that plasticizes the polymer matrix and lowers the barrier for translational hops. It is noted that *E*_a_ reduces from ≈11 kJ mol^–1^ at 1 wt % to <3 kJ mol^–1^ at 20 wt %, while *D*_0_ drops toward a plateau of ∼5 × 10^–9^ m^2^ s^–1^. Experimental measurements^48^ in amorphous PET films at 20–70 °C reported diffusivities slightly less than 10^–11^ m^2^ s^–1^, whereas our model, which is built on fully “preloaded” configurations, yields *D* ≈ 10^–11^–10^–10^ m^2^ s^–1^ at these temperatures. It should be noted that experimental studies of water transport in amorphous PET films under conventional vapor-sorption conditions reported substantially larger diffusion activation energies than those obtained here. For example, Burgess et al.^49^ reported an activation energy of 46.4 ± 3.0 kJ mol^–1^ for water diffusion in amorphous PET over 15–45 °C, together with strong concentration dependence and penetrant-plasticization behavior as water activity increases. The difference arises because the present simulations and thin-film sorption experiments probe different transport regimes. In our MD calculations, water molecules are already distributed throughout an amorphous PET domain at prescribed loadings, so the calculated diffusivities describe the intrinsic mobility of water within already hydrated PET. By contrast, experimental diffusivities for industrial or laboratory-prepared PET films usually reflect macroscopic transport during water uptake into initially dry or weakly hydrated materials, which includes the slower ingress through a less-plasticized matrix and may also be affected by crystallinity and processing-induced structural constraints. For this reason, the simulated activation energies are lower and the diffusivities at 20–70 °C are somewhat higher than those typically reported for dry-film sorption experiments. The loading- and temperature-dependent diffusivities reported here should therefore be interpreted as describing water mobility in prehydrated amorphous PET under reactor-relevant conditions, rather than direct absorption kinetics in initially dry macroscopic films. Under hydrothermal conditions at higher temperatures, the uptake resistance that dominates transport in initially dry films becomes less severe. Consistently, experimental permeation studies (at higher temperatures) have reported diffusivities in the 10^–10^–10^–9^ m^2^ s^–1^ range,^50^ which are in better agreement with our predictions. Under these conditions, the experimental measurements more closely reflect the intrinsic water mobility captured by our simulations.

### 3.4 Chemical Potential and Water Uptake

Whereas Section 3.3 has established how the mobility of sorbed water varies with concentration and temperature, an equally important question for process design is how much water the polymer can thermodynamically accommodate. We address this by performing Widom-insertion calculations (using the OPLS-AA force field for the PET framework and the TIP4P model for water) on equilibrium trajectories with different water preloads to obtain the water chemical potential in the polymer phase, and then determining the equilibrium uptake by identifying the loading at which this chemical potential equals the environmental chemical potential, following the procedure established by Deng et al.^13^

Figure 5a,b summarize μ_ads_ as a function of water loading (0–20 wt %) between 300 and 600 K. At each temperature, the data follows the expected logarithmic rise and is captured well by the logarithmic fit (*R* ^2^ ≥ 0.97). The thermodynamic saturation point at each temperature is defined as the intersection of the μ_ads_–loading curve and the environmental water chemical potential from Section 3.1 (red × in Figure 5a,b); this intersection corresponds to the equilibrium loading. Figure 5c shows that the average water–polymer interaction energy (blue) remains within the reported range^51,52^ of −32 to −51 kJ mol^–1^ per water molecule, changing from −51.9 kJ mol^–1^ to −48.5 kJ mol^–1^. The dispersion component (green, right axis) accounts for about 10% of the total interaction energy, consistent with previous work,^53^ and the dispersion component is much more temperature sensitive, decreasing by ∼40% from −5.6 to −3.4 kJ mol^–1^. In contrast, the electrostatic contribution, which dominates the overall attraction and includes hydrogen bonding, varies by less than 5% and becomes nearly temperature independent above 550 K (Figure S5). Across working conditions, the average water–polymer interaction energy exhibits the same nonmonotonic trend as the polymer density change (initially becoming more favorable as the matrix swells, then weakening as the polymer partially densifies) as shown in Figure 2b, indicating that swelling/free-volume evolution is the primary structural control.

**Figure 5:**
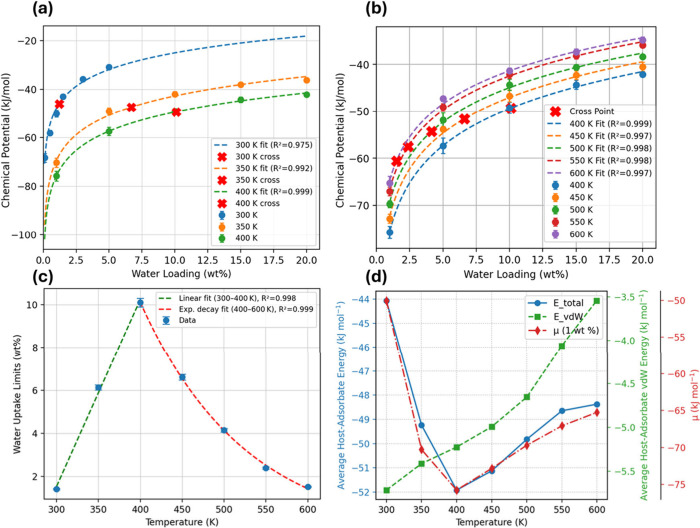
(a) Chemical potential of water in amorphous PET vs loading at 300, 350, and 400 K; dashed curves are log fits and red × indicate successive-temperature intersection points. (b) Same analysis for 400–600 K. (c) Temperature dependence of average host–adsorbate interaction energy and μ at 1 wt % loading. (d) Water-uptake limit derived from (a, b): linear rise between 300–400 K and exponential decay from 400–600 K.

Figure 5d shows that the maximum sorption capacity increases almost linearly from ∼1 wt % at 300 K to ≈10 wt % at 400 K, then drops exponentially to <2 wt % by 600 K. This nonmonotonic trend reflects the competition between free-volume creation, which enhances uptake below the glass-transition region (Figure S3), and the rapid loss of PET–water cohesion at higher temperatures. Together with the logarithmic μ_ads_–loading fits, these saturation limits define the physically accessible hydration states during hydrothermal processing and set the initial conditions for the reactive-MD hydrolysis simulations discussed next.

### 3.5 ReaxFF Simulation

Building on the water uptake capacity established above, ReaxFF MD simulations at different degrees of swelling are employed at 1200 K to probe the initial chain-breaking process as an accelerated degradation approach, with the focus on comparing the influence of water uptake on the reaction rate. Figure 6 compares three amorphous PET cells that begin with 1, 5, or 10 wt % water. The number-average molecular weight (Figure 6a) for all systems falls logarithmically but with markedly different slopes: notably, Mw drops by barely 15% in 2 ns at 1 wt % water, by ∼45% at 5 wt %, and by more than 60% at 10 wt %. The strong loading dependence confirms that each additional water molecule accelerates ester hydrolysis.

**Figure 6:**
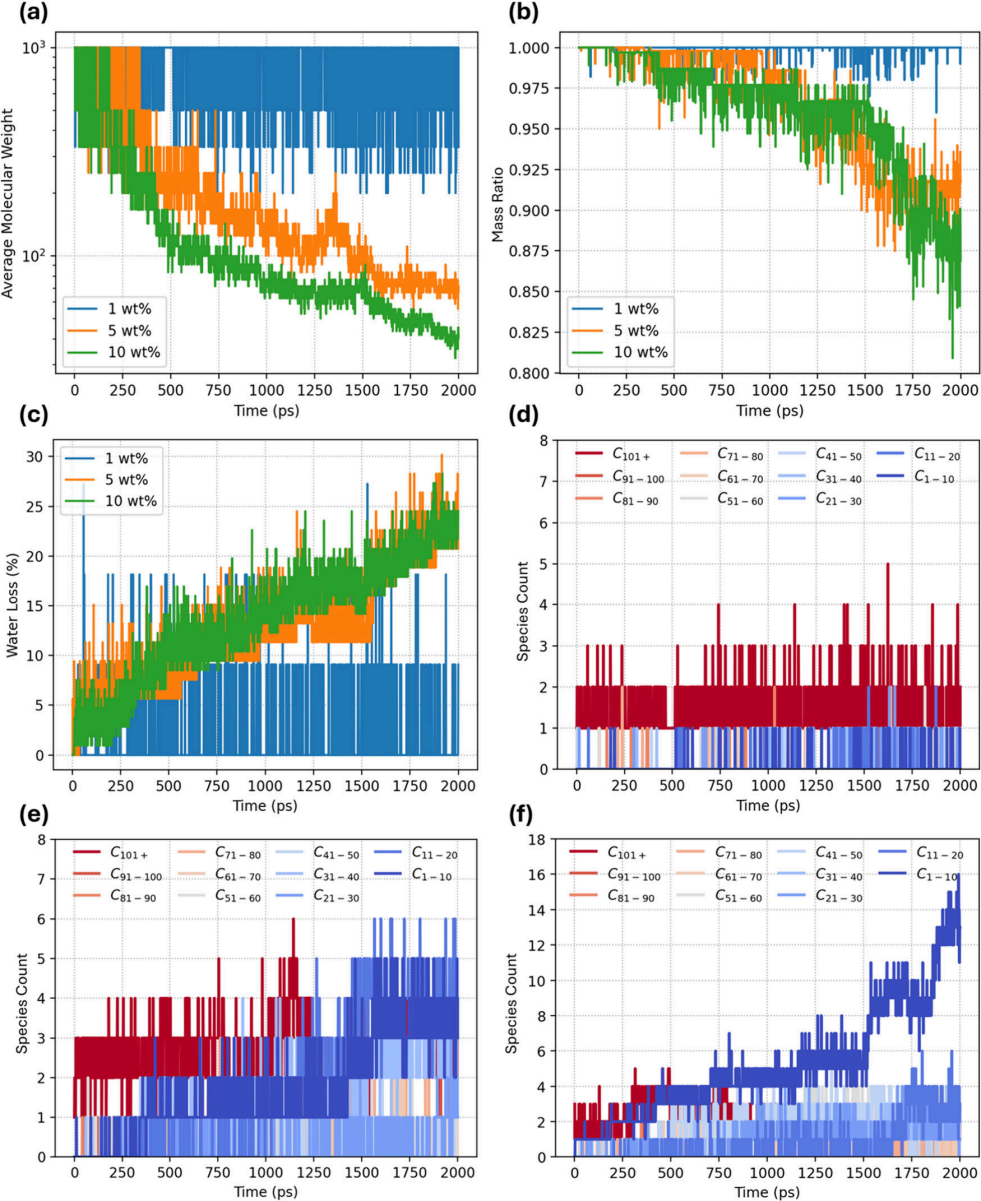
Time evolution of PET hydrolysis at 1200 K for initial water loadings of 1, 5, and 10 wt %: (a) logarithmic drop in average molecular weight, (b) remaining polymer mass fraction, and (c) cumulative water mass lost to the gas phase. Corresponding fragment-size distributions, color-coded from long chains (C_101+_, red) to small oligomers/monomers (C_1–10_, dark blue), for (d) 1 wt %, (e) 5 wt %, and (f) 10 wt %.

Figure 6b recasts the same trajectories as residual polymer mass, counting every fragment shorter than 20 carbons as mass loss, roughly the size below which chains behave as soluble oligomers under experimental hydrolysis conditions. With this threshold, the 10 wt % system exceeds 10% mass loss within the first nanosecond, whereas the 1 wt % system never reaches 2% over the full run, underscoring the water-limited nature of early degradation. Figure 6c tracks the fraction of the original water inventory consumed. The curves climb only when Mw is already falling, and the fractional water loss scales with the degree of chain breaking, demonstrating that the ReaxFF MD simulation represents hydrolysis rather than a separate pyrolytic pathway.

Figure 6d–f merges these observations into fragment-length spectra. At 1 wt % (Figure 6d) the distribution is dominated by long chains and contains almost no sub-C_10_ species, indicating that hydrolysis events are isolated and random with low frequency. At 5 wt % (Figure 6e) a broader band of midsized oligomers appears, signaling the onset of chain peeling as the water content increases. At 10 wt % (Figure 6f), fragments smaller than ten carbon atoms proliferate sharply after ∼1.5 ns, marking a regime in which further breakdown of trimers and dimers peeled from long polymer chains occurs. Together, these results locate a practical crossover near 5 wt % water: below it, scission remains sporadic and water-limited; above it, a water-triggered cascade rapidly depolymerizes PET. For a more comprehensive view of the reaction landscape, the 10 wt % simulation is extended to 10 ns and will be discussed in the following part of this section.

Figure 7a shows two observables: the blue curve on the left axis is the cumulative fraction of water molecules that have participated in hydrolysis, and the red curve on the right axis is the number-average molecular weight (*M*_w_). Within the first 0.5 ns almost 3.5% of the initial water charge is consumed, and *M*_w_ plunges from ∼1000 to <200, confirming that ester cleavage, not thermal chain scission, dominates even at 1200 K. A more gradual water-consumption stage from 0.5 to 4 ns coincides with the disappearance of long chains and the emergence of midsized oligomers, reflecting the higher local water density required to hydrolyze newly exposed ester bonds. Beyond 6 ns the water curve levels off at ∼55% reacted, and *M*_w_ asymptotically approaches ∼50. This plateau shows that access to free water, rather than backbone stability, ultimately limits the reaction: cleaving each additional ester bond demands not just the stoichiometric 1:1 water-to-ester ratio, but a sufficiently hydrated microenvironment.

**Figure 7:**
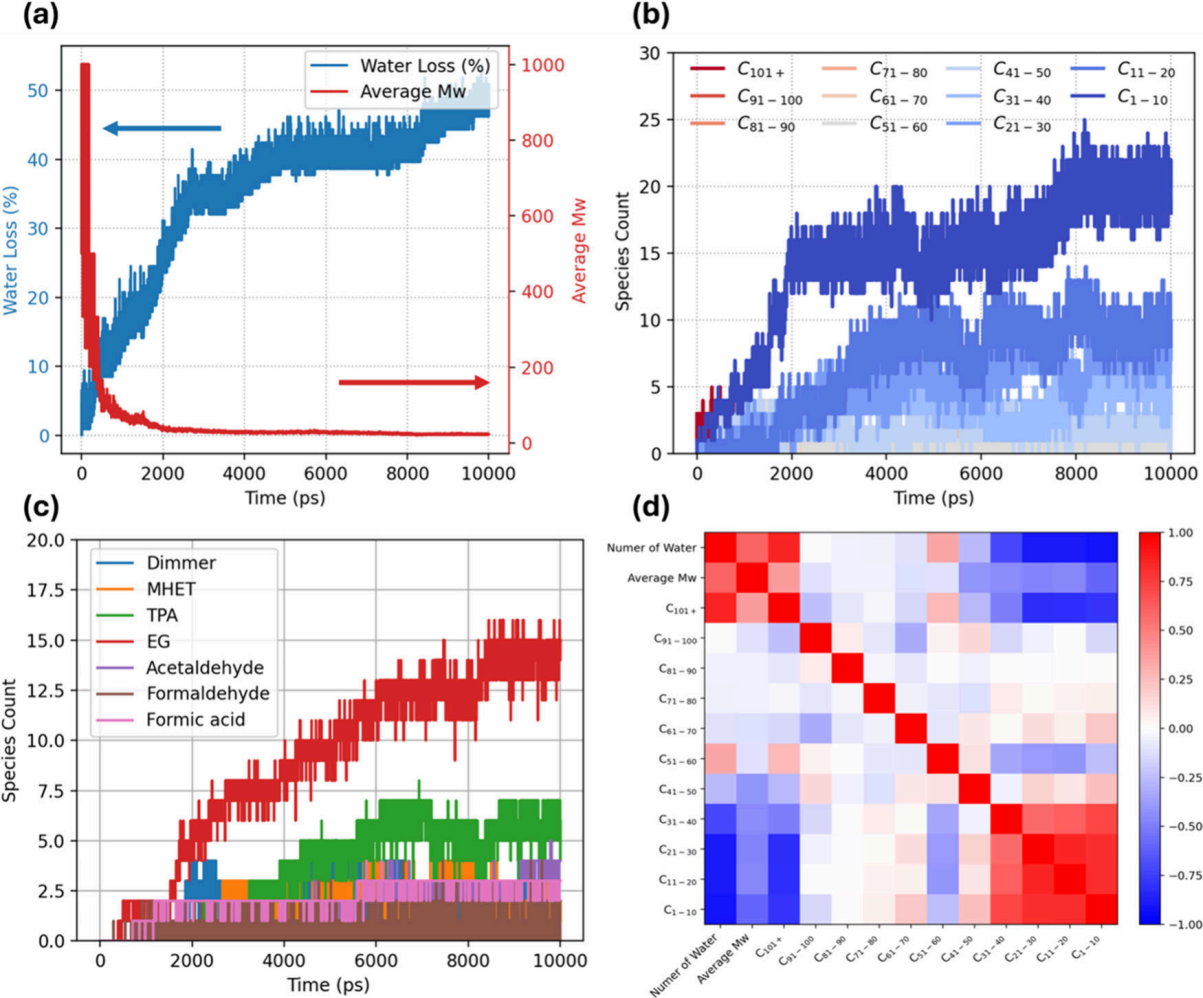
Hydrolysis dynamics and fragment evolution from extended ReaxFF MD simulation with 10 wt %: (a) Time evolution of water consumption (water loss, left axis) and number-average molecular weight (right axis) over a 10 ns trajectory. (b) Shift of the fragment-size distribution with time, grouped by total carbon count. (c) Speciated products versus time: MHET, TPA (terephthalic acid), EG (ethylene glycol), and minor carbonyls (acetaldehyde, formaldehyde, formic acid); counts rise as scission proceeds. (d) Pearson correlation matrix across the time-series variables.

The fragment statistics in Figure 7b chart the same process at the molecular level. Chains longer than 100 carbons disappear within 0.8 ns. Midsized oligomers (C_31–50_) bloom while water is still plentiful, then dwindle as they are peeled into smaller fragments. The C_1–10_ pool expands continuously until water is spent, stabilizing at ∼25 species, which matches the stoichiometric expectation for complete cleavage of the initial long chain into predominantly dimers and monomers. In Figure 7c, the product evolution is resolved by species. Ethylene glycol (EG) rises most rapidly, reaching ∼15 molecules by 10 ns, which indicates that a fraction of repeat units undergoes complete cleavage to release free EG. In contrast, most scission events populate partially depolymerized fragments, with MHET as the dominant intermediate rather than immediate monomer formation. MHET increases early and peaks near 5 ns, then decreases as it is further hydrolyzed to terephthalic acid (TPA) with the concomitant production of an additional equivalent of EG. After the free water is consumed, this secondary conversion becomes water-limited: MHET plateaus and persists alongside the accumulated TPA, consistent with kinetic arrest of the second step. Finally, aldehydes remain at trace levels and appear primarily after water depletion, suggesting they arise from secondary thermal degradation rather than the hydrolysis pathway.

Figure 7d distills the 10 ns trajectory into a Pearson correlation heatmap. *M*_w_ is strongly anticorrelated with both reacted-water fraction and the C_1–10_ fragment pool, confirming that polymer depolymerization accelerates precisely when water is being consumed and monomers proliferate. Residual (unreacted) water is positively correlated with midsized oligomers (C_21–50_) but negatively with the smallest fragments, capturing the transient “peeling” stage before water becomes scarce. Along the fragment–fragment block, adjacent size classes are mildly positive. In contrast, distant classes are sharply negative, indicating pathways for direct conversion of long chains into short species rather than always involving an intermediate accumulation. Although our model tracks a limited number of cells over nanosecond time scales, thus highlighting local rather than large-scale transport, the results still demonstrate that PET depolymerization at 1200 K is clearly water-limited. Matrix loosening does create transient water-rich pockets; yet, each additional ester cleavage continues to require a highly hydrated microenvironment, far exceeding the simple 1:1 water-to-ester stoichiometry. It should be noted that, under the conditions of ReaxFF MD simulations used in this work to explore a more hydrolysis-dominant regime, the primary reactions observed are ester-bond cleavage, chain scission, and the formation of smaller hydrolysis or decomposition products. Within the simulated time scale, neither significant aromatic ring opening nor extensive oxygen functionalization of long-chain fragments is observed. In contrast, studies conducted under more aggressive hydrolysis conditions,^54^ particularly at very high temperatures, show that radical-driven chemistry is significantly enhanced. Under such conditions, additional reaction pathways, such as aromatic ring opening and oxygen functionalization, may become more prominent.

### 3.6 General Reaction Mechanism

Time-resolved network analysis of the 10 ns ReaxFF trajectory (1200 K, 10 wt % H_2_O) reveals a reproducible progression from sporadic scission to directed end-group peeling, with the shift governed by local hydration and the availability of free water (Figures 8 and 9). Converting configurations into 2 ns fragment-conversion graphs (Figure 9a–e) and companion Sankey maps (early 0–2 ns vs late 8–10 ns from Figure 8 and all 0–10 ns from Figure S6) allows the mechanism to be read directly from flux topology and node centrality rather than inferred from isolated events.

**Figure 8:**
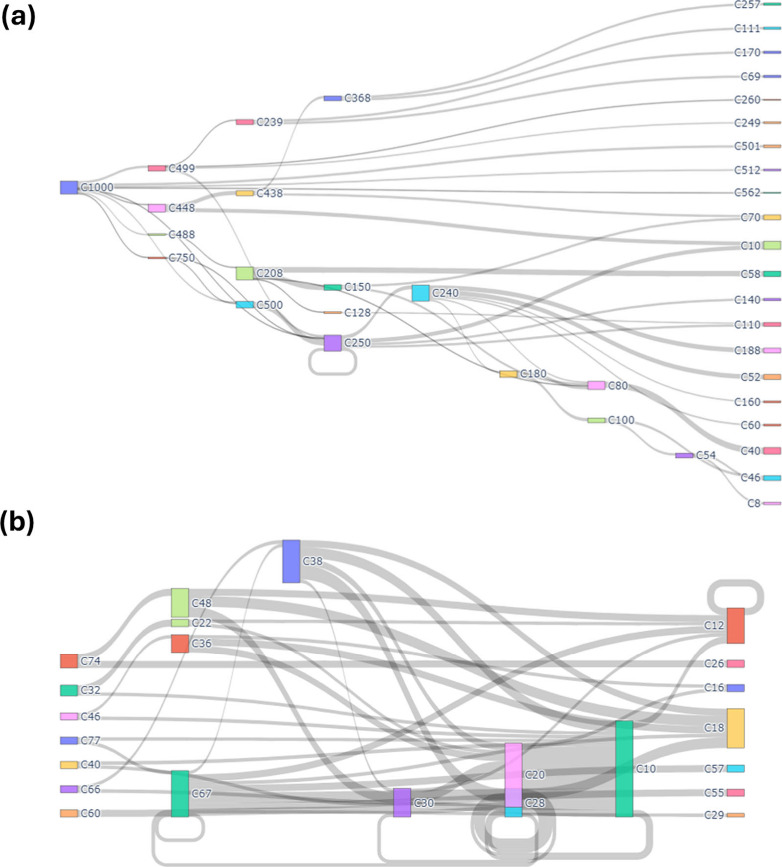
Fragmentation pathways during PET hydrolysis, early vs late stage: (a) 0–2 ns (early stage) and (b) 8–10 ns (late stage).

**Figure 9:**
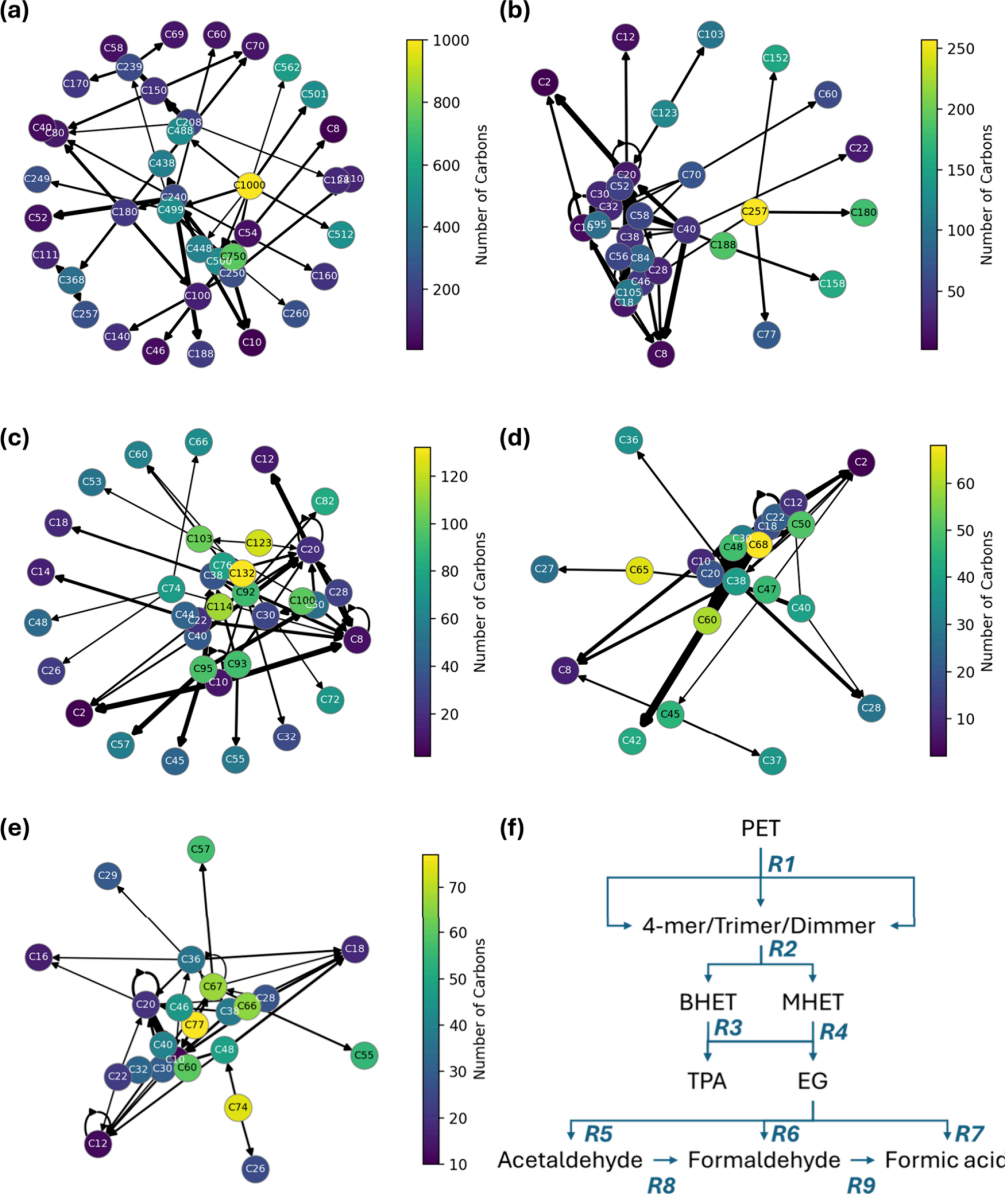
Fragment-conversion networks for PET hydrolysis at 1200 K, 10 wt % H_2_O: (a) 0–2 ns, (b) 2–4 ns, (c) 4–6 ns, (d) 6–8 ns, (e) 8–10 ns. Nodes (C#, # represents the carbon number of the species) are colored by carbon count, arrow thickness scales with reaction frequency. (f) The condensed full major reaction scheme.

At the early stage (0–2 ns), Figures 8a and 9a show that transitions are dominated by isolated ester-bond scission within very long chains, producing large oligomers (typically C_500–100_), while the majority of mass remains in high-carbon bins or recirculates via self-loops. The network at this stage is star-shaped around the long-chain species, indicating that hydrolysis happens at scattered sites and that no small-fragment sink has emerged. Occasional direct formation of EG is observed, but it accounts for a minor fraction of events, consistent with limited availability of contiguous hydrogen-bond relays and modest local water activity.

Between 2–8 ns (Figure 9b–d) the hub fragments shift downward to C_90–50_; arrows now interconnect oligomers and funnel into BHET, MHET, and TPA, indicating a short chain production stage in which water attacks newly exposed esters: BHET → TPA + EG and MHET → TPA + EG together account for∼60% of all events. After 8 ns (Figure 9e), the network contracts further: C_20–10_ nodes become the main hubs, production of TPA and EG is constrained by water availability. Formaldehyde oxidation to formic acid is the final step to emerge, but it remains a trace path. By 8–10 ns, the flux (Figure 8b) shifts strongly toward short oligomers and monomers, indicating end-initiated “peeling” and accumulation in small-size sinks with little return to larger fragments. This change in flow (from sporadic high-C splits to directed peeling) captures the chain-breaking mechanism and anticipates the observed rise in MHET/BHET → TPA + EG formation. The complete time-resolved Sankey across 0–10 ns (contiguous windows) is provided in the SI.

Taken together, these results support a three-stage mechanism: (i) random chain scission limited by water diffusion, (ii) end-group-driven peeling once carboxylic sites proliferate, and (iii) low-frequency, water-starved side reactions that generate small oxygenates. The simplified scheme in Figure 9f summarizes these findings and maps them to reactions R1–R9. Although stage (ii) consumes both ester bonds and water at a 1:1 ratio, complete depolymerization in industrial practice will require either excess liquid water or continuous water replenishment, which aligns with our simulation results showing that a higher water/PET ratio accelerates hydrolysis, as well as with previous experimental/simulation observations.^55^

### 3.7 Insights via DFT Calculations

ReaxFF-MD studies in the previous section show that rising local water content systematically accelerates PET hydrolysis, and we turn to density functional theory (DFT) to clarify the underlying mechanism changes and understand the details of the intermediates.

A PET repeat-unit trimer surrounded by *n* = 1, 2, 4, 8, and 16 explicit water molecules (details of model structures can be found in the Computational Methods section and Figure S7). Each cluster is fully optimized to emulate the bulk environment. The water-normalized stabilization metric plotted in Figure 10a is defined as shown in eq 4.

4 $$E_{\mathrm{stab}}\left(n\right)=\frac{E_{\mathrm{tot}}\left(n\right)-nE_{H_{2}O}-E_{\mathrm{PET}}}{n}$$

**Figure 10:**
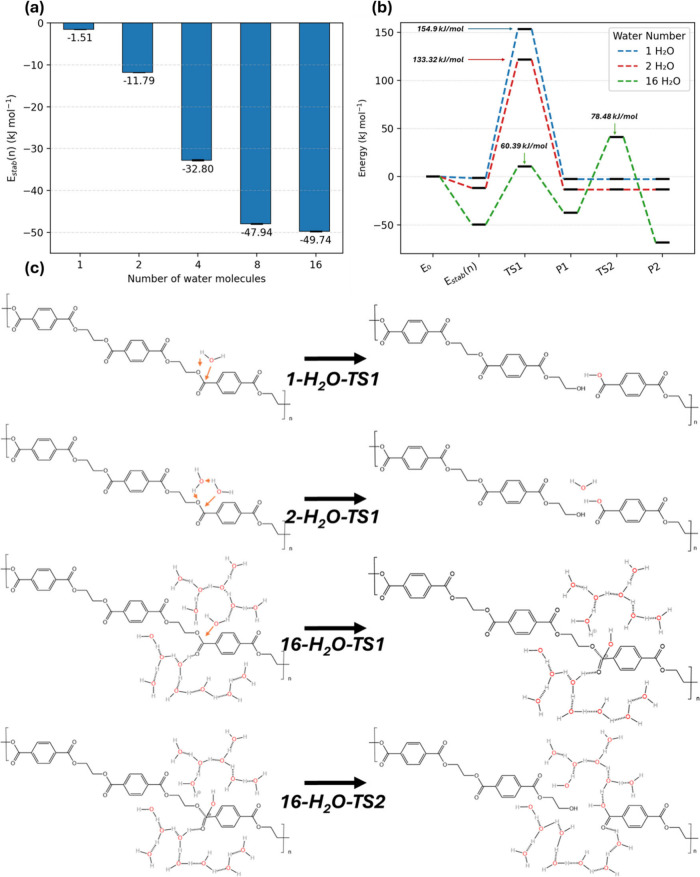
(a) Stabilization energy (*E*_stab_(*n*)) of the reactant complex as the solvation shell grows from 1 to 16 H_2_O. (b) Energy profiles for reaction processes with 1, 2, and 16 water clusters. (c) Demonstration of the structures (reactants and products) observed during the DFT calculation.

A more negative value signals a greater per-molecule capacity to prestabilize the reactant complex.

Figure 10a shows that a single water molecule offers only marginal stabilization of the prereactive complex, about – 1.5 kJ mol^–1^ per water. As further molecules crowd the carbonyl region, each contributes a larger share of stabilization until the first hydration shell closes near *n* = 8. By *n* = 16, the total gain reaches −49.8 kJ mol^–1^. The steadily more negative values confirm that an increasingly dense hydrogen-bond network “pre-organizes” the ester bond for nucleophilic attack. In addition, the observed upshift of the alkoxy-oxygen p band serves as direct electronic evidence that water-rich environments activate the ester linkage by destabilizing the alkoxy-oxygen lone pair (see Figure S8).

The reaction-coordinate diagrams in Figure 10b trace how this solvent preorganization translates into lower barriers. With only one water, the ester cleavage follows a single concerted step whose transition state lies roughly at 155 kJ mol^–1^, which is consistent with the previous DFT study of the single water-PET breaking process.^56^ Introducing a second water creates a two-water proton relay, dropping the barrier to 133 kJ mol^–1^. A qualitative change appears at *n* = 16: the dense solvent cage stabilizes a discrete tetrahedral oxyanion intermediate, so that the original concerted event splits into two sequential steps with much smaller energy hills (Δ*E* ^‡^_1_ ≈ 60 kJ mol^–1^, Δ*E* ^‡^_2_ ≈ 78 kJ mol^–1^). A bulk-like water solution, therefore, not only lowers the overall activation energy but also remodels the reaction pathway from concerted to stepwise pathways. It should be noted, however, that the 78 kJ mol^–1^ barrier from the 16-water-molecule DFT model represents the lowest barrier identified in this work, corresponding to an idealized, highly water-rich local environment with particularly favorable hydrogen-bond assistance. This value is therefore not expected to apply to all hydrolysis sites in amorphous PET. Because the polymer is heterogeneous and the local water environment varies with position and uptake, the representative barrier for hydrolysis is higher than this ideal minimum. As shown in Figure S2, the activation barriers are approximately 100–130 kJ mol^–1^ over water uptakes from 5 to 20 wt %, which is consistent with our previous computational and experimental analysis.^22^

The representative geometries in Figure 10c illustrate the structural origin of this behavior. The monohydrate transition state forces a strained four-center arrangement in which proton transfer and C–O bond scission occur simultaneously. While larger water clusters stabilize the oxyanion intermediate, the reaction can pause at substable intermediates before a second proton shuffle completes the bond cleavage. This observation agrees with previous theoretical predictions of PET–water configurations in which water surrounds the chains^57^ and provides additional structural and quantitative evidence. At low to moderate water activities, the reaction network can be treated as a single, concerted event. However, under near-saturated conditions, the two-step mechanism enabled by the tetrahedral intermediate becomes increasingly competitive and ultimately dominant. This shift closes the gap between the higher barriers (over 155 kJ mol^–1^) reported in earlier DFT work^56^ and the lower apparent activation energies from experiments and MD work.^22^

### 3.8 Insights from the Kinetic Model

A kinetic model is established that integrates reaction and diffusion across temperature and hydration, providing a clear physical picture of PET hydrolysis. First, the effective activation barrier decreases monotonically with water uptake (Figure 11a), so any operating change that raises water activity—such as different temperature and pressure that lead to greater water–polymer ratio—pushes the system to wetter states and lowers the effective activation energy. Because uptake itself is a thermodynamic function of *T* and *P*, the apparent “temperature effect” on the barrier is largely mediated by swelling. Second, the map of kinetic constants with Thiele isolines (Figure 11b) shows ϕ ≪ 1 over the entire feasible space for a 1 mm slab model, implying η ≈ 1 and thus reaction-controlled kinetics. In other words, internal diffusion is not the rate-limiting step under the dimensions and conditions considered; only at much larger characteristic lengths does diffusion-limited (or mixed) behavior become relevant.

**Figure 11:**
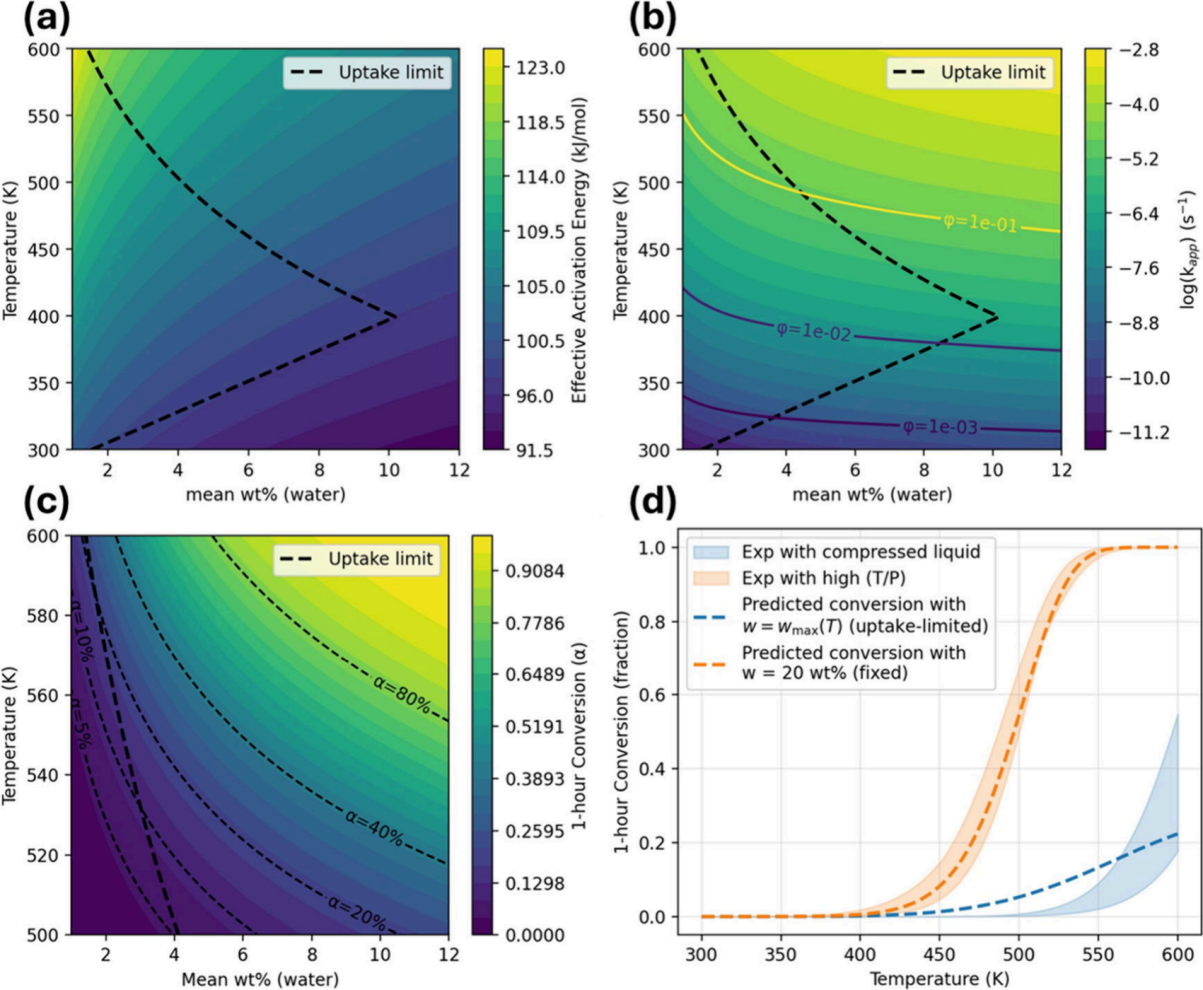
Model results for PET neutral hydrolysis. (a) Effective activation energy and (b) kinetic constants with Thiele isolines. (c) Predicted 1 h conversion with iso-α contours (20%, 40%, and 80%). (d) One hour conversion vs temperature along the uptake limit (blue) and at fixed 20 wt % (orange), overlaid with literature ranges.

The conversion maps translate these trends into outcomes. Along the uptake-limited path (dashed curves), the predicted 1 h conversion remains modest because attainable hydration is capped (Figure 11c). At higher mean loadings (e.g., 20 wt %), conversion rises sharply, consistent with the barrier drop. Notably, the model embeds local heterogeneity: a small fraction of active wet microdomains can pull down the effective activation energy through log-sum-exp averaging, so rates can exceed what one would infer from the mean water uptake alone. This reconciles the model with experiments from both high-*T*/*P* data sets^58,59^ and lower *T* with compressed-liquid case^55^ (Figure 11d). The high-temperature and pressure data sets show substantially higher 1 h conversion than would be possible if PET water content is constrained to the solid-state uptake limit *w*_max_(*T*). In the model, that behavior is reproduced only when water availability is not limiting, represented by the fixed *w* = 20 wt % curve, which effectively captures a plasticized or partially molten polymer phase with elevated water activity. In contrast, the compressed liquid measurements at lower temperatures fall near the uptake-limited trajectory, consistent with PET remaining largely solid and water access being governed by sorption and diffusion constraints.

Overall, the model shows how multiscale simulations can be incorporated into a realistic kinetic description and indicates that PET hydrolysis in water is chemistry-limited under practical geometries. Raising water activity by increasing temperature or pressure, using excess water, or applying pretreatments that enhance swelling and thereby increase the fraction of active wet domains emerges as the most effective way to accelerate conversion.

## 4 Conclusions

Our work links reactor conditions to molecular reactivity, yielding a coherent picture of PET depolymerization in hot, pressurized water. Translating temperature–pressure to the water chemical potential establishes the polymer-phase uptake limit and generates μ-consistent swollen configurations, from which density and diffusion follow. Across the accessible range, modest increases in hydration produce disproportionate changes in free volume and mobility. Once water is absorbed and clustered, transport accelerates, and the system enters a regime where chemistry, rather than access, becomes the rate-controlling factor. Time-resolved reactive simulations, read through fragment-conversion networks and Sankey maps, show a reproducible progression from random, water-limited scission of long chains to end-group-driven peeling that funnels through BHET/MHET and terminates in TPA and EG. Electronic-structure calculations rationalize this crossover: extended hydrogen-bond relays stabilize the tetrahedral intermediate and lower the activation energy barrier relative to isolated water attack, providing a mechanistic basis for the observed hydration threshold. Embedding the loading-dependent diffusivity and DFT-anchored barriers in a kinetic model reproduces the acceleration under high water activity and the slowdown as water is consumed, clarifying when uptake, transport, or chemistry governs the overall rate.

These results point to practical levers for hydrothermal recycling. Maintaining polymer-phase hydration above the threshold via pressure control, liquid-water contact, or active replenishment can promote peeling and shorten the time for degradation, allowing local water depletion to trap the system in short-oligomer basins despite elevated temperatures. Because ester cleavage can be accelerated by a surrounding water network, stoichiometric supply, and mass transport at the polymer–water interface, these factors are as decisive as intrinsic barriers. The density map ρ­(*w*, *T*, *p*) and the μ-based uptake framework provide readily measurable or inferable state variables that can anchor scale-up models and process control. Overall, a self-consistent multiscale framework is established that provides a quantitatively predictive description of PET hydrolysis under working conditions. The same logic and many of the parameters are transferable to related polyesters, offering a blueprint for designing tunable, high-efficiency depolymerization processes grounded in molecular-level insight.

## Supplementary Material



## Notes

Supplementary figures and discussion present (i) the *T*–*p* curve via the Antoine equation, (ii) details of the kinetic model, (iii) the transition temperature, (iv) radial distribution functions at different temperatures, (v) host–adsorbate interaction energies, (vi) the reaction process diagram, (vii) PET–water structures, and (viii) the density of states (PDF)

## References

[1] Gao Z., Ma B., Chen S., Tian J., Zhao C. (2022). Converting Waste PET
Plastics into Automobile Fuels and Antifreeze Components. Nat. Commun..

[2] Geyer R., Jambeck J. R., Law K. L. (2017). Production, Use,
and Fate of All
Plastics Ever Made. Sci. Adv..

[3] Bezeraj E., Debrie S., Arraez F. J., Reyes P., Van Steenberge P. H.
M., D’hooge D. R., Edeleva M. (2025). State-of-the-Art of
Industrial PET Mechanical Recycling: Technologies, Impact of Contamination
and Guidelines for Decision-Making. RSC Sustain.

[4] Rahimi A., García J. M. (2017). Chemical
Recycling of Waste Plastics for New Materials
Production. Nat. Rev. Chem..

[5] Umdagas L., Orozco R., Heeley K., Thom W., Al-Duri B. (2025). Advances in
Chemical Recycling of Polyethylene Terephthalate (PET) via Hydrolysis:
A Comprehensive Review. Polym. Degrad. Stab..

[6] Hong H., Ki D., Seo H., Park J., Jang J., Kim K.-J. (2023). Discovery
and Rational Engineering of PET Hydrolase with Both Mesophilic and
Thermophilic PET Hydrolase Properties. Nat.
Commun..

[7] Dubelley F., Planes E., Bas C., Pons E., Yrieix B., Flandin L. (2018). Predictive Durability of Polyethylene Terephthalate
toward Hydrolysis over Large Temperature and Relative Humidity Ranges. Polymer.

[8] Woodard L. N., Grunlan M. A. (2018). Hydrolytic Degradation and Erosion
of Polyester Biomaterials. ACS Macro Lett..

[9] Thomsen T. B., Almdal K., Meyer A. S. (2023). Significance of Poly­(Ethylene Terephthalate)
(PET) Substrate Crystallinity on Enzymatic Degradation. New Biotechnol.

[10] Sammon C., Mura C., Yarwood J., Everall N., Swart R., Hodge D. (1998). FTIR–ATR Studies of the Structure
and Dynamics of Water Molecules
in Polymeric Matrixes. A Comparison of PET and PVC. J. Phys. Chem. B.

[11] Arhant M., Le Gall M., Le Gac P.-Y., Davies P. (2019). Impact of Hydrolytic
Degradation on Mechanical Properties of PET - Towards an Understanding
of Microplastics Formation. Polym. Degrad. Stab..

[12] Wang X. (2016). Review of
Characterization Methods for Water-Soluble Polymers Used in Oil Sand
and Heavy Oil Industrial Applications. Environ.
Rev..

[13] Deng X., Han Y., Lin L.-C., Ho W. S. W. (2022). Computational Prediction of Water
Sorption in Facilitated Transport Membranes. J. Phys. Chem. C.

[14] Wei X., Zheng W., Sun W., Zhao L. (2024). Modeling the Enhanced
Swelling Behaviors of Poly­(Ethylene Terephthalate) Glycolysis with
Mixed EG/CHDM Using Experiments and Molecular Dynamics Simulation. Ind. Eng. Chem. Res..

[15] Hörstermann H., Hentschke R., Amkreutz M., Hoffmann M., Wirts-Rütters M. (2010). Predicting
Water Sorption and Volume Swelling in Dense Polymer Systems via Computer
Simulation. J. Phys. Chem. B.

[16] Chen Y.-R., Liou K.-H., Kang D.-Y., Chen J.-J., Lin L.-C. (2018). Investigation
of the Water Adsorption Properties and Structural Stability of MIL-100­(Fe)
with Different Anions. Langmuir.

[17] Datar A., Witman M., Lin L.-C. (2021). Monte Carlo Simulations for Water
Adsorption in Porous Materials: Best Practices and New Insights. AIChE J..

[18] Shih S.-M., Lin L.-C. (2025). Water Adsorption in Metal–Organic Frameworks:
Characteristics, Mechanisms, and Structure–Property Relationships. J. Am. Chem. Soc..

[19] Kulasinski K., Guyer R., Derome D., Carmeliet J. (2015). Water Diffusion
in Amorphous Hydrophilic Systems: A Stop and Go Process. Langmuir.

[20] Senftle T. P., Hong S., Islam M. M., Kylasa S. B., Zheng Y., Shin Y. K., Junkermeier C., Engel-Herbert R., Janik M. J., Aktulga H. M., Verstraelen T., Grama A., van Duin A. C. T. (2016). The ReaxFF Reactive Force-Field:
Development, Applications and Future Directions. Npj Comput. Mater..

[21] Ma S. M., Zou C., Bakshi B. R., Lin L.-C. (2024). Molecular
Dynamics Simulation of
Zeolite-Assisted Pyrolysis of Polystyrene: Material Selection and
Mechanistic Insights. Ind. Eng. Chem. Res..

[22] Ma S. M., Pereira P., Pester C. W., Savage P. E., Bakshi B. R., Lin L.-C. (2025). Understanding
PET Hydrolysis via Reactive Molecular
Dynamics Simulation and Experimental Investigation. J. Phys. Chem. B.

[23] Fayon P., Devémy J., Emeriau-Viard C., Ballerat-Busserolles K., Goujon F., Dequidt A., Marty A., Hauret P., Malfreyt P. (2023). Energetic
and Structural Characterizations of the PET–Water
Interface as a Key Step in Understanding Its Depolymerization. J. Phys. Chem. B.

[24] Pan J. (2021). Scaling up
System Size in Materials Simulation. Nat. Comput.
Sci..

[25] Bell I. H., Wronski J., Quoilin S., Lemort V. (2014). Pure and Pseudo-Pure
Fluid Thermophysical Property Evaluation and the Open-Source Thermophysical
Property Library CoolProp. Ind. Eng. Chem. Res..

[26] Thomson G.
Wm (1946). The Antoine
Equation for Vapor-Pressure Data. Chem. Rev..

[27] Peng D.-Y., Robinson D. B. (1976). A New Two-Constant Equation of State. Ind. Eng. Chem. Fundam..

[28] Thompson A. P., Aktulga H. M., Berger R., Bolintineanu D. S., Brown W. M., Crozier P. S., in ’t
Veld P. J., Kohlmeyer A., Moore S. G., Nguyen T. D., Shan R., Stevens M. J., Tranchida J., Trott C., Plimpton S. J. (2022). LAMMPS
- a Flexible Simulation Tool for Particle-Based Materials Modeling
at the Atomic, Meso, and Continuum Scales. Comput.
Phys. Commun..

[29] Zhang W., van Duin A. C. T. (2018). Improvement of the ReaxFF Description for Functionalized
Hydrocarbon/Water Weak Interactions in the Condensed Phase. J. Phys. Chem. B.

[30] Gittus O. R., Bresme F. (2021). Thermophysical Properties
of Water Using Reactive Force
Fields. J. Chem. Phys..

[31] Jewett A. I., Stelter D., Lambert J., Saladi S. M., Roscioni O. M., Ricci M., Autin L., Maritan M., Bashusqeh S. M., Keyes T., Dame R. T., Shea J.-E., Jensen G. J., Goodsell D. S. (2021). Moltemplate: A Tool
for Coarse-Grained Modeling of
Complex Biological Matter and Soft Condensed Matter Physics. J. Mol. Biol..

[32] Sanches N. B., Dias M. L., Pacheco E. B. A. V. (2005). Comparative
Techniques for Molecular
Weight Evaluation of Poly­(Ethylene Terephthalate) (PET). Polym. Test..

[33] Dhaka V., Singh S., Anil A. G., Sunil Kumar Naik T. S., Garg S., Samuel J., Kumar M., Ramamurthy P. C., Singh J. (2022). Occurrence, Toxicity and Remediation
of Polyethylene Terephthalate
Plastics. A Review. Environ. Chem. Lett..

[34] Dubbeldam D., Calero S., Ellis D. E., Snurr R. Q. (2016). RASPA: Molecular
Simulation Software for Adsorption and Diffusion in Flexible Nanoporous
Materials. Mol. Simul..

[35] Witman M., Ling S., Jawahery S., Boyd P. G., Haranczyk M., Slater B., Smit B. (2017). The Influence
of Intrinsic Framework
Flexibility on Adsorption in Nanoporous Materials. J. Am. Chem. Soc..

[36] Bin Y., Oishi K., Yoshida K., Matsuo M. (2004). Mechanical Properties
of Poly­(Ethylene Terephthalate) Estimated in Terms of Orientation
Distribution of Crystallites and Amorphous Chain Segments under Simultaneous
Biaxially Stretching. Polym. J..

[37] Sangkhawasi M., Remsungnen T., Vangnai A. S., Poo-arporn R. P., Rungrotmongkol T. (2022). All-Atom Molecular Dynamics Simulations on a Single
Chain of PET and PEV Polymers. Polymers.

[38] Mochizuki K., Sumi T., Koga K. (2016). Liquid–Liquid
Phase Separation
of N-Isopropylpropionamide Aqueous Solutions above the Lower Critical
Solution Temperature. Sci. Rep..

[39] Stenberg S., Stenqvist B. (2020). An Exact Ewald
Summation Method in Theory and Practice. J.
Phys. Chem. A.

[40] Yagasaki T., Matubayasi N. (2025). Molecular
Dynamics Simulations of Concentrated and
Dilute Aqueous Solutions of Poly­(N-Isopropylacrylamide) Using a Modified
OPLS-AA Model. J. Phys. Chem. B.

[41] Kühne T. D., Iannuzzi M., Del Ben M., Rybkin V. V., Seewald P., Stein F., Laino T., Khaliullin R. Z., Schütt O., Schiffmann F., Golze D., Wilhelm J., Chulkov S., Bani-Hashemian M. H., Weber V., Borštnik U., Taillefumier M., Jakobovits A. S., Lazzaro A., Pabst H., Müller T., Schade R., Guidon M., Andermatt S., Holmberg N., Schenter G. K., Hehn A., Bussy A., Belleflamme F., Tabacchi G., Glöß A., Lass M., Bethune I., Mundy C. J., Plessl C., Watkins M., VandeVondele J., Krack M., Hutter J. (2020). CP2K: An Electronic
Structure and Molecular Dynamics Software Package - Quickstep: Efficient
and Accurate Electronic Structure Calculations. J. Chem. Phys..

[42] Paier J., Hirschl R., Marsman M., Kresse G. (2005). The Perdew–Burke–Ernzerhof
Exchange-Correlation Functional Applied to the G2–1 Test Set
Using a Plane-Wave Basis Set. J. Chem. Phys..

[43] Grimme S., Antony J., Ehrlich S., Krieg H. (2010). A Consistent and Accurate
Ab Initio Parametrization of Density Functional Dispersion Correction
(DFT-D) for the 94 Elements H-Pu. J. Chem. Phys..

[44] Hartwigsen C., Goedecker S., Hutter J. (1998). Relativistic Separable Dual-Space
Gaussian Pseudopotentials from H to Rn. Phys.
Rev. B.

[45] VandeVondele J., Hutter J. (2007). Gaussian Basis Sets for Accurate
Calculations on Molecular
Systems in Gas and Condensed Phases. J. Chem.
Phys..

[46] Henkelman G., Uberuaga B. P., Jónsson H. (2000). A Climbing Image Nudged Elastic Band
Method for Finding Saddle Points and Minimum Energy Paths. J. Chem. Phys..

[47] Okuwaki K., Mochizuki Y., Doi H., Kawada S., Ozawa T., Yasuoka K. (2018). Theoretical Analyses
on Water Cluster Structures in
Polymer Electrolyte Membrane by Using Dissipative Particle Dynamics
Simulations with Fragment Molecular Orbital Based Effective Parameters. RSC Adv..

[48] Cai W. D., Ramesh N., Tihminlioglu F., Danner R. P., Duda J. L., DèHaan A. (2002). Phase Equilibrium
and Diffusion of Solvents in Polybutadiene:
A Capillary-Column Inverse Gas Chromatography Study. J. Polym. Sci., Part B: Polym. Phys..

[49] Burgess S. K., Mikkilineni D. S., Yu D. B., Kim D. J., Mubarak C. R., Kriegel R. M., Koros W. J. (2014). Water Sorption in Poly­(Ethylene Furanoate)
Compared to Poly­(Ethylene Terephthalate). Part 2: Kinetic Sorption. Polymer.

[50] Peterson R. L., Neppel E. P., Holmes D., Trinh P. A., Ofoli R. Y., Dorgan J. R. (2025). Upcycling of Waste
Poly­(Ethylene Terephthalate): Ammonolysis
Kinetics of Model Bis­(2-Hydroxyethyl Terephthalate) and Particle Size
Effects in Polymeric Substrates. ChemSusChem.

[51] Launay A., Thominette F., Verdu J. (1999). Water Sorption in Amorphous Poly­(Ethylene
Terephthalate). J. Appl. Polym. Sci..

[52] Zheng J., Wang D., Zhang Q., Song M., Jiao M., Zhang Z. (2022). Molecular Dynamics
Simulation and Structure Changes of Polyester
in Water and Non-Aqueous Solvents. Materials.

[53] Ho C.-H., Valentine M. L., Chen Z., Xie H., Farha O., Xiong W., Paesani F. (2023). Structure and Thermodynamics of Water
Adsorption in NU-1500-Cr. Commun. Chem..

[54] Liu X., Wang T., Chu J., He M., Li Q., Zhang Y. (2020). Understanding Lignin
Gasification in Supercritical Water Using Reactive
Molecular Dynamics Simulations. Renew. Energy.

[55] Pereira P., Savage P. E., Pester C. W. (2023). Neutral
Hydrolysis of Post-Consumer
Polyethylene Terephthalate Waste in Different Phases. ACS Sustain. Chem. Eng..

[56] Huang J., Xu W., Long Y., Zhu Y., Chen S., Duan W., Ou J., Wang H., Dong C., Tian S. (2024). Studies on Hydrolysis/Alcoholysis/Ammonolysis
Mechanisms of Ethylene Terephthalate Dimer Using DFT Method. Arab. J. Chem..

[57] Conroy S., Zhang X. (2024). Theoretical Insights into Chemical
Recycling of Polyethylene Terephthalate
(PET). Polym. Degrad. Stab..

[58] Stanica-Ezeanu D., Matei D. (2021). Natural Depolymerization
of Waste Poly­(Ethylene Terephthalate) by
Neutral Hydrolysis in Marine Water. Sci. Rep..

[59] Mishra S., Zope V. S., Goje A. S. (2003). Kinetics
and Thermodynamics of Hydrolytic
Depolymerization of Poly­(Ethylene Terephthalate) at High Pressure
and Temperature. J. Appl. Polym. Sci..

