# Supplementary material for: Water-Network-Triggered Breakdown: Multiscale Theoretical Insights into PET Hydrolysis under Working Conditions
Source: J Phys Chem B. 2026 Apr 28;130(18):4911–27. doi: 10.1021/acs.jpcb.6c00486 (PMC13158921; doi:10.1021/acs.jpcb.6c00486)
Supplement: Supplementary file 1 [file jp6c00486_si_001.pdf]

Supporting Information

For

Water-Network-Triggered Breakdown: Multiscale Theoretical Insights into PET Hydrolysis under Working Conditions

Shuangxiu Max Ma[a], Changlong Zou[a], Bhavik R Bakshi[b,c,d*], Li-Chiang Lin[a,e,*]

[a] William G. Lowrie Department of Chemical and Biomolecular Engineering, The Ohio State University, Columbus, Ohio 43210, United States

[b] School for Engineering of Matter, Transport and Energy, Arizona State University, Tempe, AZ 85281, United States

[c] School of Sustainability, Arizona State University, Tempe, AZ 85281, United States

[d] School of Complex Adaptive Systems, Arizona State University, Tempe, AZ 85281, United States

[e] Department of Chemical Engineering, National Taiwan University, Taipei 106319, Taiwan

***** Corresponding authors: B.R. Bakshi (bhavik.bakshi@asu.edu) and L.-C. Lin (lclin@ntu.edu.tw)

Contents

1. **T-P curve via Antoine equation** 3
2. **Details for kinetic model** 4
3. **Transition temperature** 7
4. **RDF of water at different temperatures** 9
5. **Host–adsorbate interaction energy** 10
6. **Reaction diagram** 11
7. **PET-water structures** 13
8. **The density of states** 15

1. T-P curve via Antoine equation

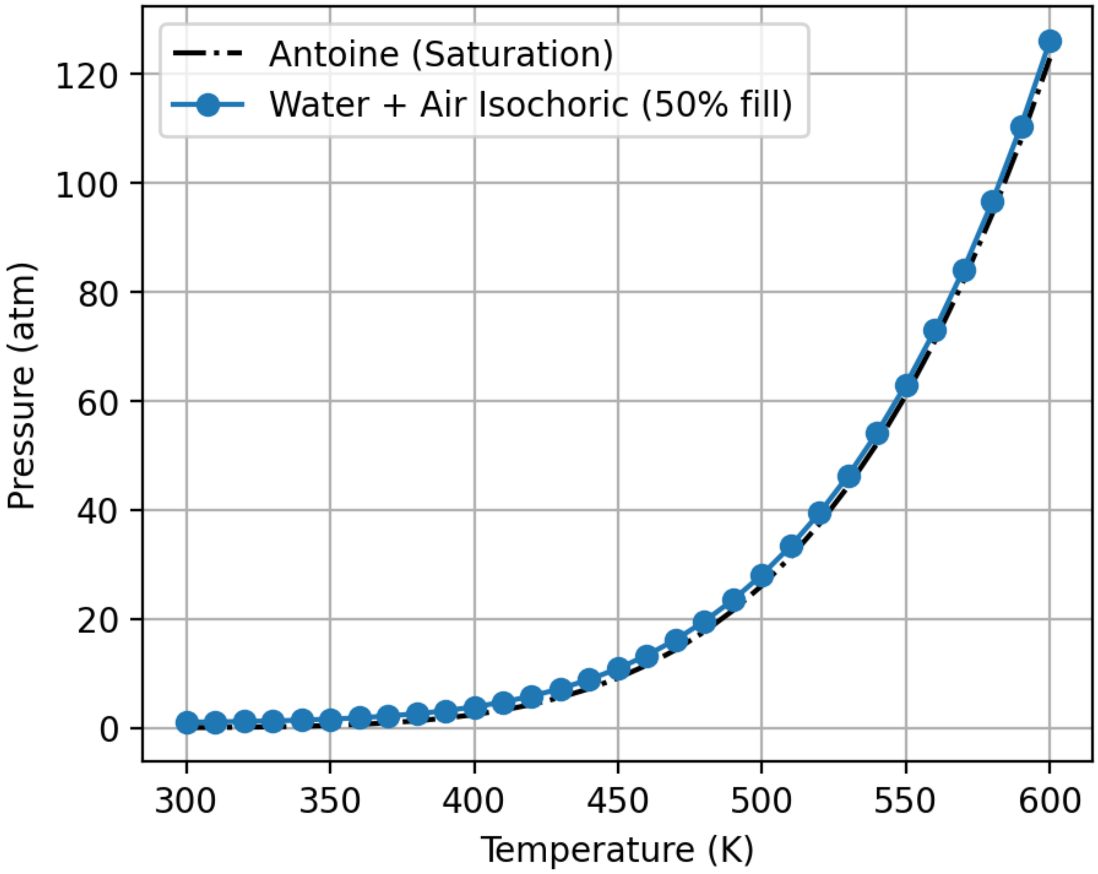

Figure S1. Temperature–pressure relationship for liquid water in a sealed autoclave. The dashed black line is the saturated-vapor pressure of pure water calculated from the Antoine equation, while solid blue markers trace the isochoric pressure calculated from CoolProp.[1]

The classical Antoine equation is used to calculate the saturation vapor pressure of water as a function of temperature:

$$log10(P_{mmHg}) = A - \frac{B}{C + T(°C)}$$

A = 8.14019, B = 1810.94, and C = 244.485, with T (°C) = T (K) – 273.15. These constants, taken from fits to experimental data (NIST, 0–300 °C), yield the vapor pressure P_{mmHg}, which can be converted to other units (e.g., atm in this case) as needed. This method effectively assumes a large headspace so that the water vapor pressure equals its saturation value, thereby neglecting any effect of non-condensable gases, as shown in Figure S1.

2. Details for kinetic model

We model the neutral hydrolysis of PET in water with a reaction–diffusion framework. Intrinsic kinetics depend on local hydration and temperature, while internal mass transfer is captured through a Thiele-modulus effectiveness factor. On a (T, $\bar{w}$) grid (T in K; mean water loading $\bar{w}$ in wt%), we compute: (i) the effective activation barrier E_{eff} (T, $\bar{w}$), (ii) the apparent first-order rate constant k_{app} (T, $\bar{w}$), and (iii) the 1-h conversion α (T, $\bar{w}$; t = 3600 s). We also evaluate the maximum attainable conversion along an uptake-limit. Unless stated otherwise, energies are in kJ mol^{-1} (R = 0.008314 kJ mol^{-1} K^{-1}), diffusivities in m^2 s^{-1}, and times in seconds.

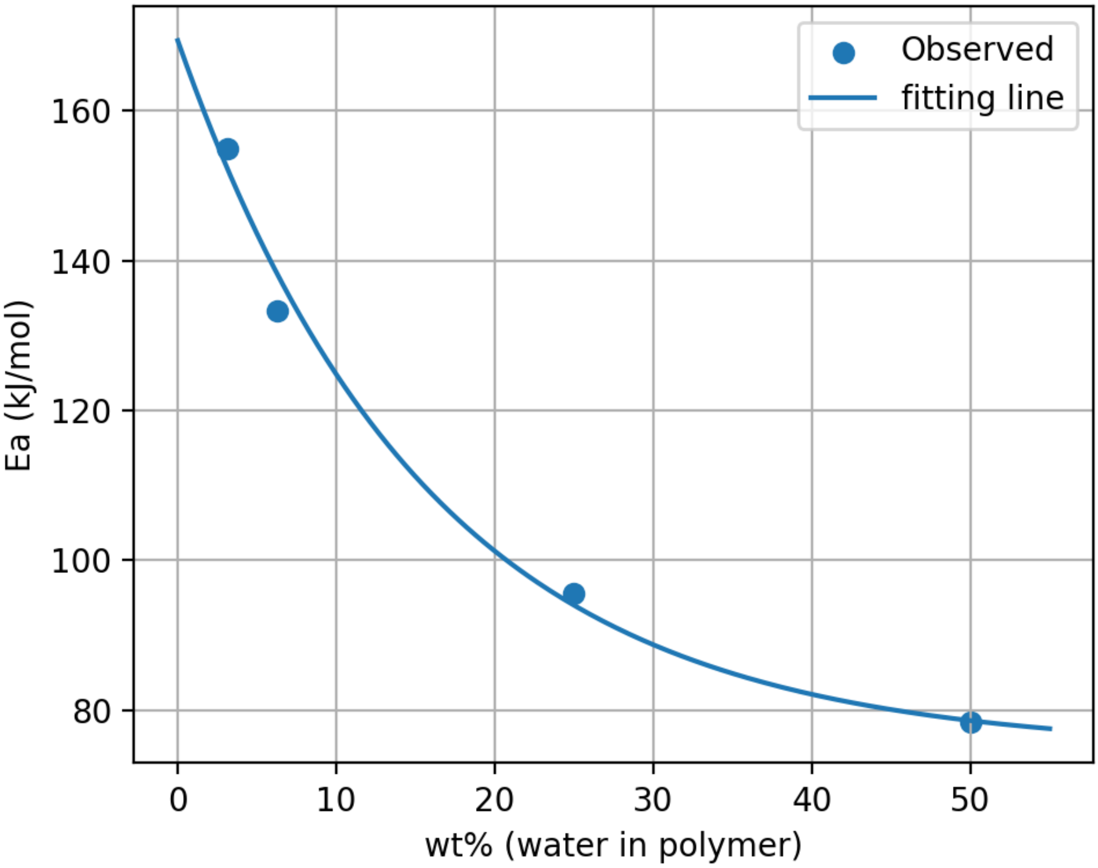

Figure S2. The activation-energy curve calculated from DFT simulations (see the details of DFT results in Section 3.7 of the main text) at different water uptakes, with the fitting line.

From fits to DFT-derived barriers (as shown in **Figure S2**), the local activation energy at hydration w (wt%) is:

$$E(w) = 74.66 + 94.72\exp(-0.063557w)\ [\ kJ/mol\ ]\ \textbf{(1)}$$

To account for spatial heterogeneity, we coarse-grain in rate space with a two-population picture: a base domain at hydration w_{base} and an active/highly swollen domain at w_{active} = 50 wt%. The mean hydration is enforced by:

$$\bar{w} = (1-f)w_{base} + f w_{active} \Rightarrow w_{base}(T,\bar{w}) = \frac{\bar{w} - f(T,\bar{w})w_{active}}{1-f(T,\bar{w})} \quad \textbf{(2)}$$

where f is the hot-domain fraction and the effective barrier is the log-sum-exp average:

$$E_{eff}(T,\bar{w}) = -RT\ln\left((1-f)e^{-E(w_{base})/(RT)} + f e^{-E(w_{active})/(RT)}\right) \textbf{(3)}$$

To reflect the empirical turn-on of wet micro-domains, we use:

$$f(T,\bar{w}) = f_{max}\left(\frac{\bar{w}}{w_{active}}\right)^n \sigma\left(\frac{T-T_p}{\Delta T}\right), \quad \sigma(x) = \frac{1}{1+e^{-x}} \quad \textbf{(4)}$$

where n = 1.5, w_{active} = 50 wt%, and ΔT = 25 K.

The intrinsic pseudo-first-order rate constant is:

$$k_{rxn}(T,\bar{w}) = A\exp\left(-\frac{E_{eff}(T,\bar{w})}{RT}\right) \textbf{(5)}$$

We use the neutral-hydrolysis pre-factor A = 2.77 × 10^6 s^{-1}. The polymer-phase diffusivity is Arrhenius in temperature with a hydration-dependent pre-factor and barrier (same as the main text):

$$D(T,\bar{w}) = D_0(\bar{w})\exp\left(-\frac{E_D(\bar{w})}{RT}\right) \quad \textbf{(6)}$$

$$E_D(\bar{w}) = E_\infty + (E_0 - E_\infty)e^{-k_{Ea}\bar{w}}, \quad D_0(\bar{w}) = D_{0,\infty} + \left(D_{0,0} - D_{0,\infty}\right)e^{-k_{D0}\bar{w}} \textbf{(7)}$$

with parameters: E_∞ = 3.2877 kJ mol^{-1}, E_0 = 12.0210 kJ mol^{-1}, k_{Ea} = 0.189952 $(wt\%)^{-1}$; $D_{0,\infty}$ = 4.6588 × 10^{-9} m^2 s^{-1}, $D_{0,0}$ = 1.2155 × 10^{-8} m^2 s^{-1}, k_{D0} = 0.26285 $(wt\%)^{-1}$. In the model, we assume a slab of half-thickness L and neglect external film resistance to represent the swollen layer. The Thiele modulus and effectiveness factors are:

$$\phi(T,\bar{w};L) = L\sqrt{\frac{k_{rxn}(T,\bar{w})}{D(T,\bar{w})}}, \quad \eta(\phi) = \frac{\tanh\phi}{\phi} \quad \textbf{(8)}$$

The apparent rate constant is:

$$k_{app}(T,\bar{w};L) = \eta(\phi)\,k_{rxn}(T,\bar{w}) \textbf{(9)}$$

To represent the hydration ceiling, we employ a piecewise fit for w_{max}(T):

$$w_{max}(T) = 0.0870T - 24.5635, \quad 300 \leq T < 400 \text{ K} \quad \textbf{(10)}$$

$$w_{max}(T) = -0.7858 + 10.9202e^{-0.0080(T-400)}, \quad 400 \leq T \leq 600 \text{ K} \quad \textbf{(11)}$$

For comparison, we convert reported Arrhenius pairs (A, E_a) into 1-h conversions and show a min–max envelope for several neutral-hydrolysis data sets from high temperatures/pressure operating conditions and a regular compressed water case under lower temperature in sealed reactors. We also show a band from Pereira et al.[2] using A = 2.77×10^6 s^{-1} and E_a = 123 ± 10 kJ mol^{-1}.

3. Transition temperature

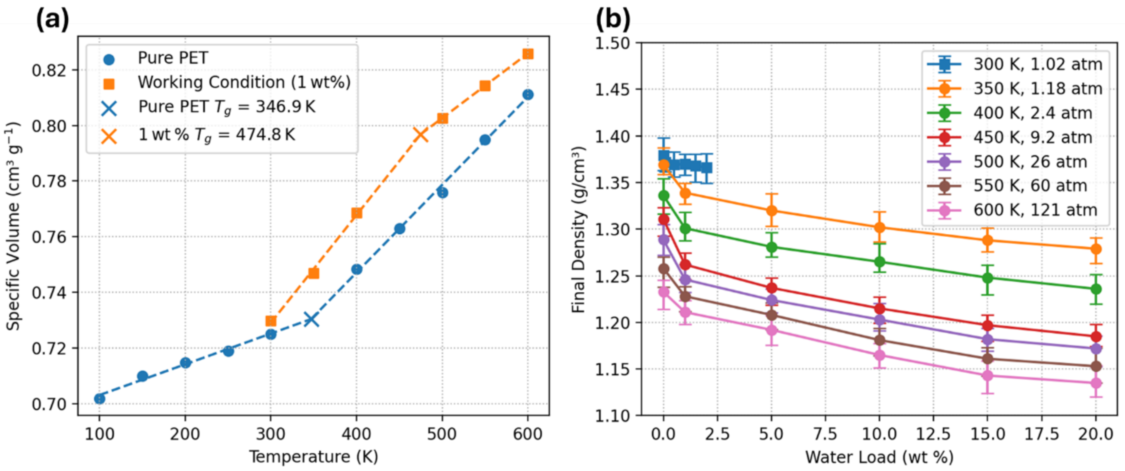

Figure S3. **(a)** Specific volume of amorphous PET as a function of temperature for the dry polymer (blue circles) and for PET pre-loaded with 1 wt% water under the working-condition chemical potential (orange squares). Dashed lines are linear fits to the glassy and rubbery regions; their intersection gives the glass-transition temperature T_g. **(b)** Results of PET density versus water uptake.

Figure S3a tracks the specific volume v =1/ρ of the amorphous PET model as temperature is ramped from 100 to 600 K under zero external pressure. For the dry reference (blue circles) the v(T) curve shows the familiar bilinear trend: a shallow thermal-expansion slope below the glass transition and a steeper slope in the rubbery regime. Linear regression of the two regions yields a glass-transition temperature T_g = 346.9 K, in line with experimental and simulated values reported for quenched, water-free PET (342–356 K),[3] which also confirms the good accuracy of the force field we use. When the polymer matrix is pre-equilibrated with 1 wt% water at the chemical potential corresponding to our hydrothermal "working condition". Along the operational T–p ramp (reaching ~120 atm at 600 K) the 1 wt%-hydrated structure exhibits an apparent T_g ~130 K higher as an effect of pressure-suppressed volume expansion, not of water-induced stiffening. Because this shifted T_g lies well above our lowest simulation temperature (300 K), two notable changes occur: first, the specific volume in the glassy region is already ~0.5 % higher than the dry baseline, reflecting the free-volume contribution of interstitial water molecules; second, the slope break that defines T_g shifts dramatically upward to 474.8 K. The elevated transition temperature indicates that, under high-activity water, the polymer backbone becomes stiffer rather than plasticized; this stiffening arises because the internal pressure increases along the isochoric T–p path as the temperature rises, effectively preloading the network. Since the "working-condition" trajectory follows this coupled T–p curve, the resulting

value should be regarded as an effective, path-dependent glass transition rather than the traditional atmospheric-pressure T_g. While **Figure S3b** shows that the water uptake inside the PET polymer can reduce the density from the dry state by 10% at most. In addition, at a fixed temperature/pressure set, the density decrease is approximately linear in water loading over 1–20 wt%, indicating that each additional wt% of sorbed water creates a roughly proportional amount of extra free volume.

4. RDF of water at different temperatures

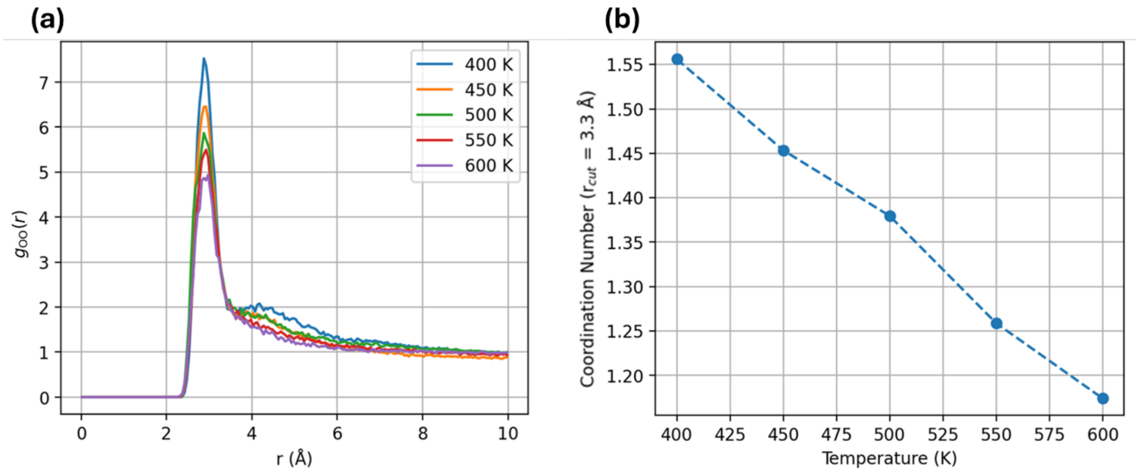

Figure S4. **(a)** O–O radial distribution functions of absorbed water in PET (1 wt %) at 400–600 K. **(b)** First-shell coordination number (r_{cut} = 3.3 Å) extracted from **(a)** decreases monotonically with temperature, evidencing progressive disruption of hydrogen-bonded clusters.

Figure S4 quantifies how the hydrogen-bond network of adsorbed water evolves between 400 K and 600 K. The oxygen–oxygen radial distribution functions $g_{OO}(r)$ (**Figure S4a**) retain a distinct first-shell peak at r ≈ 2.8 Å—characteristic of hydrogen bonding—but the peak height falls steadily as temperature rises, while the subsequent minimum becomes shallower. The coordination in **Figure S4b** drops from 1.55 at 400 K to 1.18 at 600 K. Even the low-temperature value is far below the bulk-water benchmark of ~4.4, confirming that confinement within the amorphous free volume fragments the network into dimers and small oligomers rather than extended clusters. The monotonic decline of n_{OO} highlights a progressive loss of hydrogen-bond partners with heating, a structural change that both reduces the cohesive energy density of the absorbed phase and contributes to the rising diffusivity reported in Section 3.3.

5. Host–adsorbate interaction energy

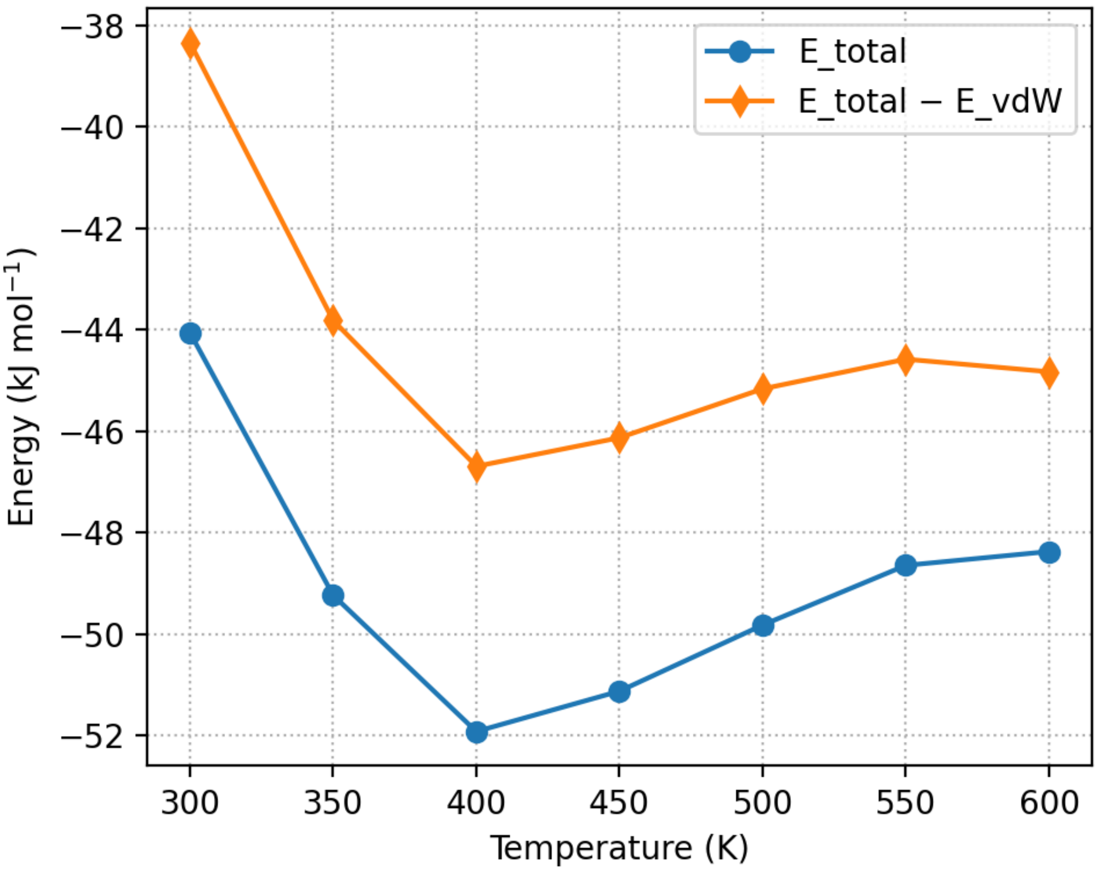

Figure S5. Temperature dependence of the host–adsorbate interaction energy for 1 wt % water in amorphous PET.

Figure S5 dissects the PET–water interaction enthalpy over 300–600 K for the 1 wt % system. The full average energy of absorbed water (E_{total}, blue) deepens from –44 kJ mol^{-1} at 300 K to a minimum of –52 kJ mol^{-1} near 400 K, then relaxes monotonically to –48 kJ mol^{-1} by 600 K. Subtracting the London-dispersion component isolates the electrostatic/H-bond term (E_{total}–E_{vdW}, orange), which displays the same non-monotonic profile but shifted upward by ≈6 kJ mol^{-1} as a temperature-dependent dispersion contribution. The energy drop towards 400 K coincides with the change in chemical potential. Beyond this temperature, the drop of London-dispersion leads to the change of total interaction energy and thus weakens the net binding; the concurrent drop in cohesive energy density facilitates the rise in water diffusivity. The ratio of the van-der-Waals term (10% of the total energy) underscores that the observed trends are dominated by coulombic energetics rather than by dispersion interactions with the polymer matrix.

6. Reaction diagram

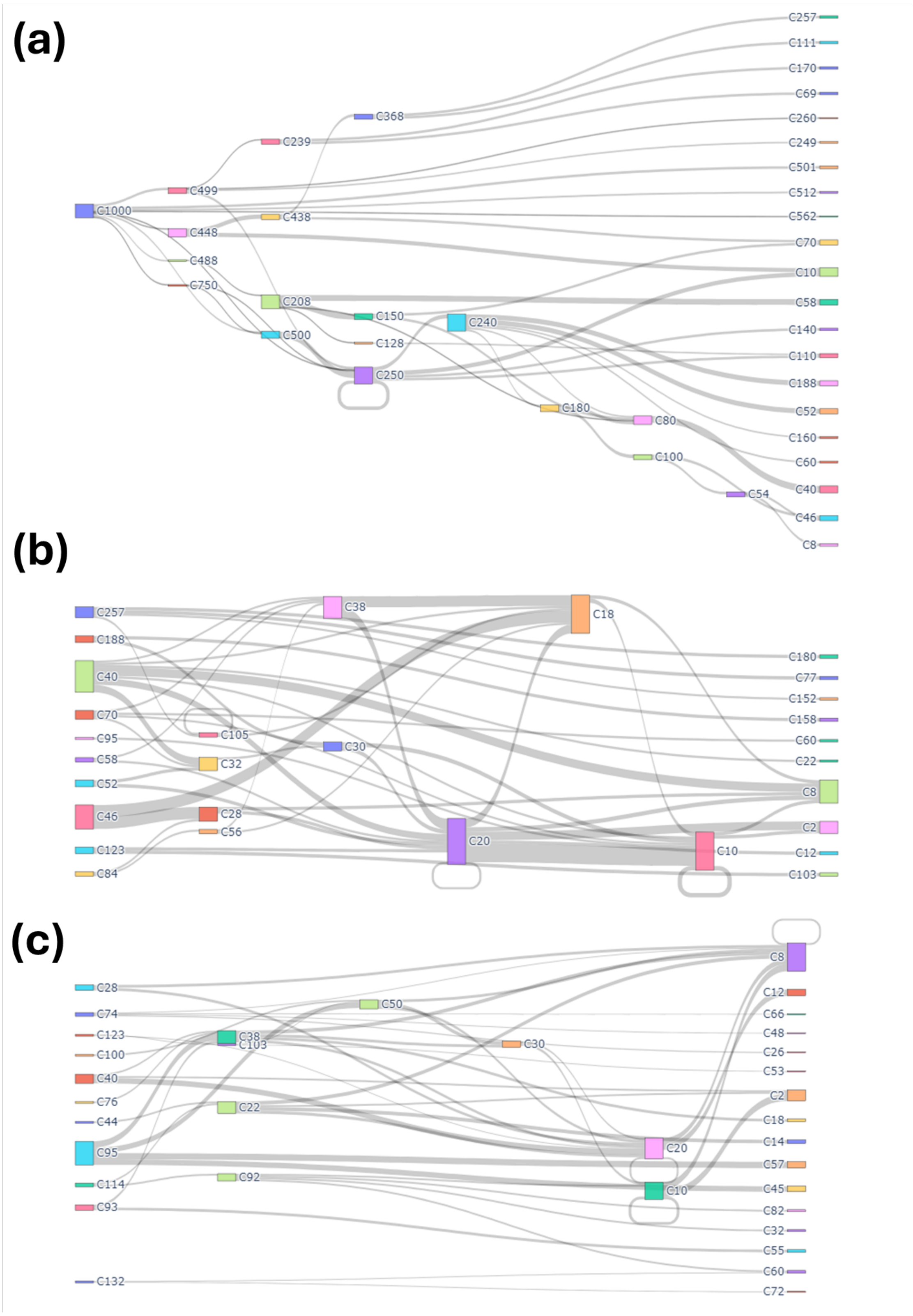

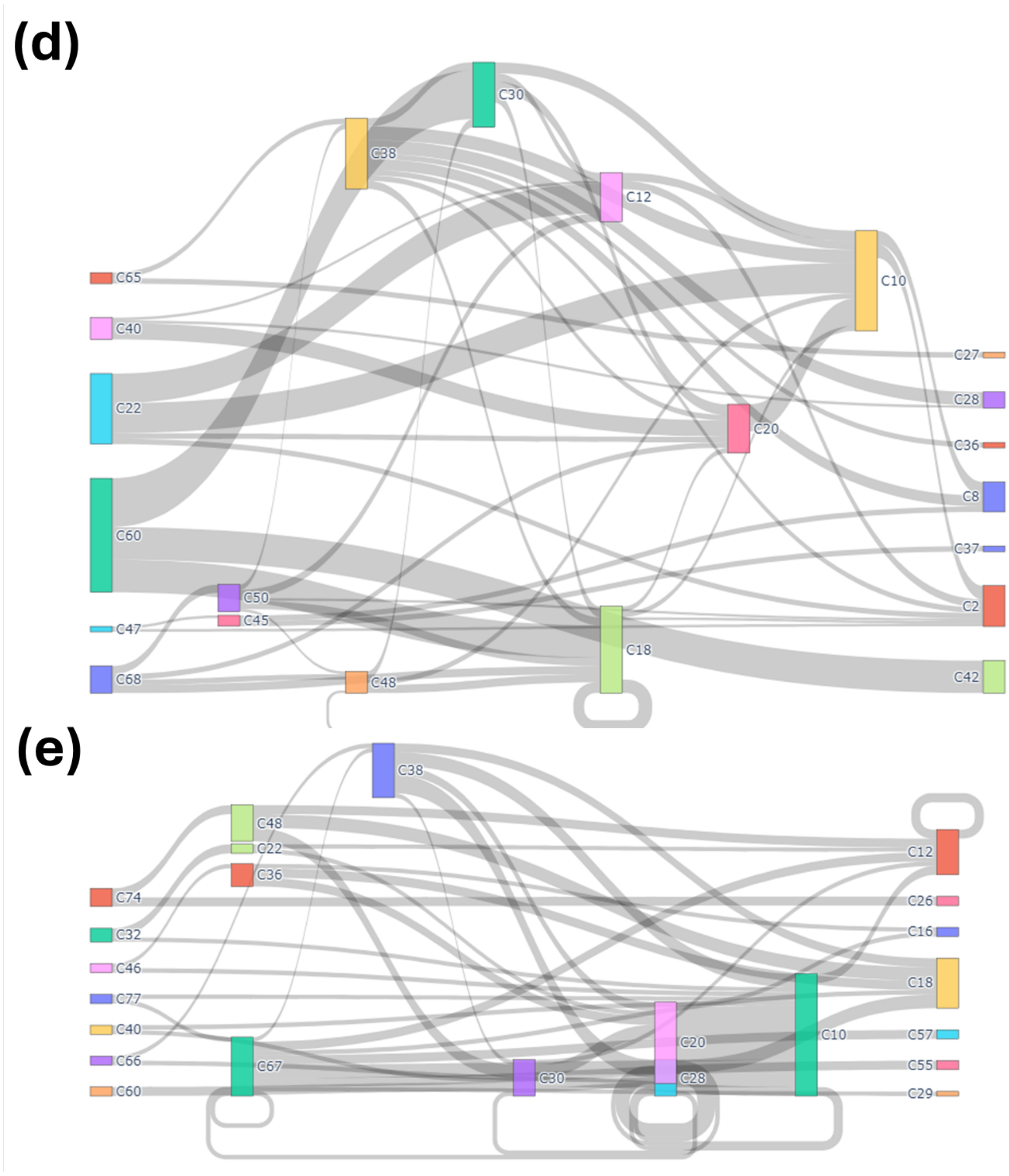

Figure S6. Fragmentation pathways during PET hydrolysis, early vs. late stage. **(a)** 0–2 ns (early stage), **(b)** 2-4 ns, **(c)** 4-6 ns, **(d)** 6-8 ns, and **(e)** 8–10 ns (end stage).

Time-resolved Sankey diagrams constructed from the 10 ns ReaxFF trajectory at 1200 K with 10 wt% H_2O exhibit a repeatable progression: early, sporadic chain scissions give way to end-group peeling. The timing of this shift correlates with local hydration and the availability of free water, and the 2 ns windowed representations (**Figure S6**) allow the mechanism to be read directly from flux topology and node centrality measures rather than inferred from isolated reactions.

7. PET-water structures

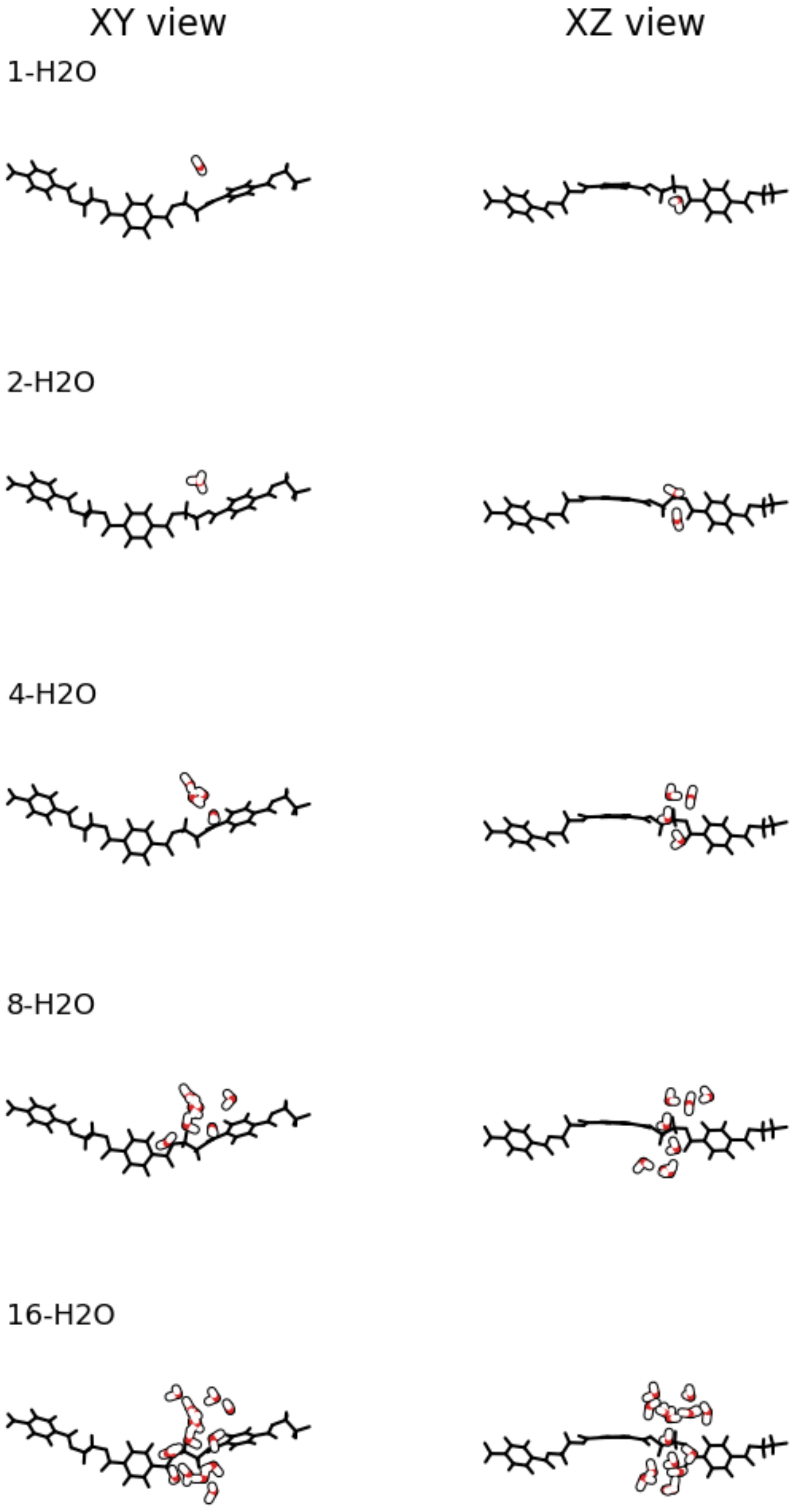

Figure S7. Orthogonal projections (XY, left; XZ, right) of representative polymer–water configurations with N = 1, 2, 4, 8, and 16 H_2O (rows). Water is rendered as ball-and-stick (O, red; H, white with black edges); non-water atoms are black sticks.

Figure S7 illustrates how water populates the local environment of a single PET chain as the number of molecules increases from 1 to 16. At very low loading (1–2 H_2O), only one or two isolated molecules adsorb at specific ester or aromatic sites along the chain, indicating a few preferred binding pockets rather than uniform coverage. At intermediate loading (4–8 H_2O), the water molecules begin to cluster in the concave region of the chain, forming small hydrogen-bonded groups that remain confined near the polymer surface rather than spreading into the surrounding free volume. At the highest loading (16 H_2O), a larger, more continuous water cluster develops around the same central segment of the chain, highlighting the tendency for hydration to remain localized in grooves and interstitial pockets instead of forming a bulk-like network. The orthogonal XY and XZ views confirm that this clustering is three-dimensional, emphasizing that even at the highest loading considered, the water molecules are still strongly coordinated to the polymer backbone.

8. The density of states

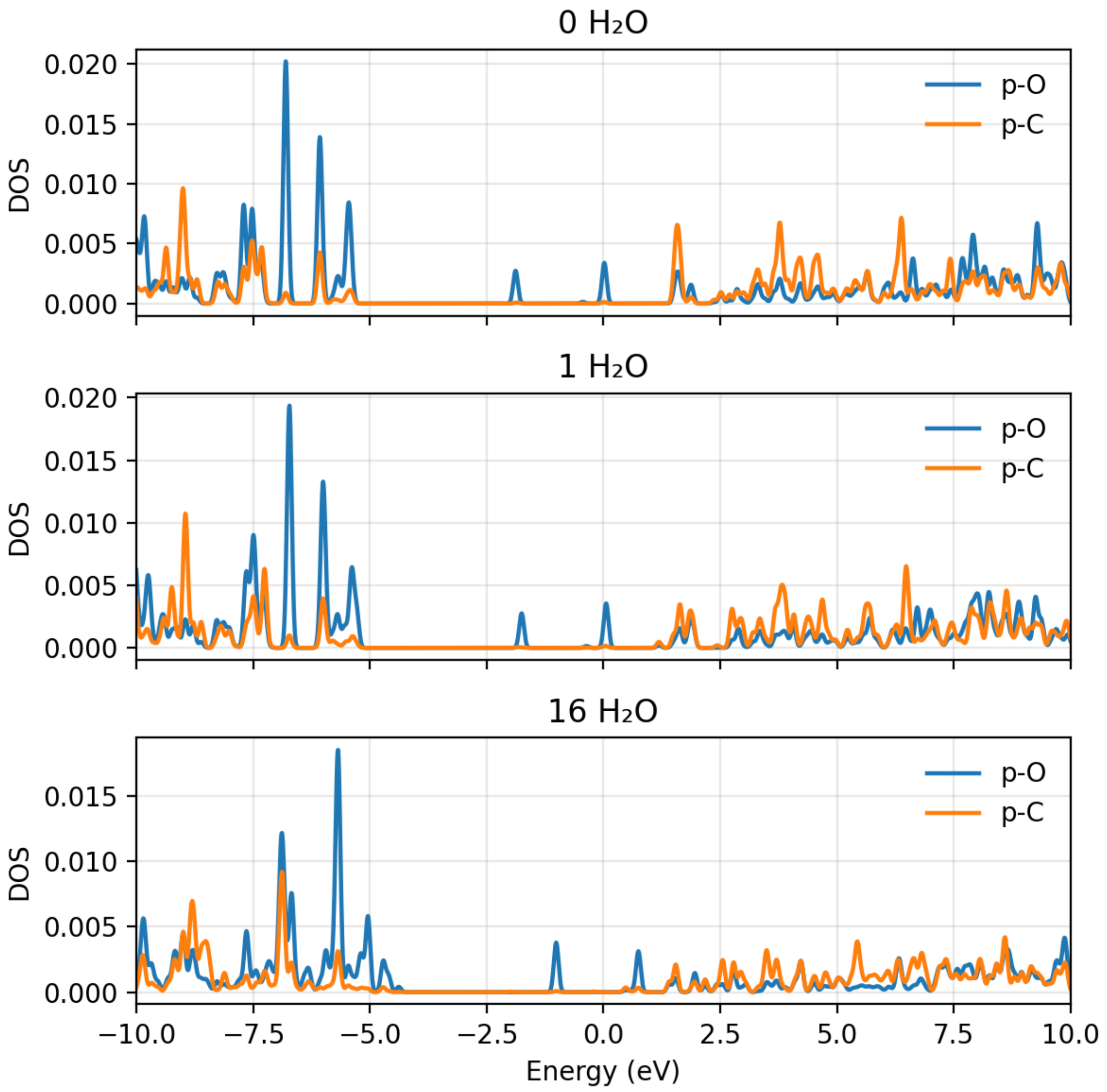

Figure S8. Projected density of states (PDOS) for the *p* orbitals of aliphatic chain carbons (*p-C*, orange) and alkoxy oxygens in ester linkers (*p-O*, blue). Top to bottom: dry model (0 H_2O), lightly hydrated model (1 H_2O), and highly hydrated model (16 H_2O). Spectra are aligned at the Fermi level (E = 0); DOS is given in arbitrary units.

To probe how hydration electronically activates the ester linkage, we examine the PDOS of the key atoms in **Figure S8**. Adding a single water molecule produces only a marginal change in the alkoxy-oxygen p band, indicating that an isolated hydrogen bond does not significantly perturb the C–O bond. In contrast, a water shell (16 H_2O) shifts this band upward to about –5.5 eV, closer to the Fermi level, indicating reduced stabilization of the O-centered lone pair. Because the C–O σ∗ antibonding level is much less affected, this hydration-induced shift narrows the gap between the filled lone-pair (n) orbital and the empty σ∗ orbital, enhancing n→σ∗ donation and

weakening the C–O bond. This provides direct electronic evidence that water-rich environments activate the ester linkage and facilitate hydrolytic cleavage.

Reference

(1) Bell, I. H.; Wronski, J.; Quoilin, S.; Lemort, V. Pure and Pseudo-Pure Fluid Thermophysical Property Evaluation and the Open-Source Thermophysical Property Library CoolProp. *Ind. Eng. Chem. Res.* **2014**, *53* (6), 2498–2508. https://doi.org/10.1021/ie4033999.

(2) Pereira, P.; Savage, P. E.; Pester, C. W. Neutral Hydrolysis of Post-Consumer Polyethylene Terephthalate Waste in Different Phases. *ACS Sustain. Chem. Eng.* **2023**, *11* (18), 7203–7209. https://doi.org/10.1021/acssuschemeng.3c00946.

(3) Sangkhawasi, M.; Remsungnen, T.; Vangnai, A. S.; Maitarad, P.; Rungrotmongkol, T. Prediction of the Glass Transition Temperature in Polyethylene Terephthalate/Polyethylene Vanillate (PET/PEV) Blends: A Molecular Dynamics Study. *Polymers* **2022**, *14* (14), 2858. https://doi.org/10.3390/polym14142858.
